# Cardiovascular disease and mortality after breast cancer in postmenopausal women: Results from the Women’s Health Initiative

**DOI:** 10.1371/journal.pone.0184174

**Published:** 2017-09-21

**Authors:** Na-Jin Park, Yuefang Chang, Catherine Bender, Yvette Conley, Rowan T. Chlebowski, G. J. van Londen, Randi Foraker, Sylvia Wassertheil-Smoller, Marcia L. Stefanick, Lewis H. Kuller

**Affiliations:** 1 School of Nursing, University of Pittsburgh, Pittsburgh, Pennsylvania, United States of America; 2 Department of Neurological Surgery, School of Medicine, University of Pittsburgh, Pittsburgh, Pennsylvania, United States of America; 3 Los Angeles Biomedical Research Institute at Harbor-UCLA Medical Center, Torrance, California, United States of America; 4 Department of Medicine, University of Pittsburgh, Pittsburgh, Pennsylvania, United States of America; 5 Division of Epidemiology, College of Public Health, The Ohio State University, Columbus, Ohio, United States of America; 6 Department of Epidemiology and Population Health, Albert Einstein College of Medicine, Bronx, New York, United States of America; 7 Stanford Prevention Research Center, Department of Medicine, Stanford University, Stanford, California, United States of America; 8 Department of Epidemiology, Graduate School of Public Health, University of Pittsburgh, Pittsburgh, Pennsylvania, United States of America; Medizinische Universitat Innsbruck, AUSTRIA

## Abstract

**Background:**

Cardiovascular disease (CVD) is the leading cause of morbidity and mortality among older postmenopausal women. The impact of postmenopausal breast cancer on CVD for older women is uncertain. We hypothesized that older postmenopausal women with breast cancer would be at a higher risk of CVD than similar aged women without breast cancer and that CVD would be a major contributor to the subsequent morbidity and mortality.

**Methods:**

In a prospective Women’s Health Initiative study, incident CVD events and total and cause-specific death rates were compared between postmenopausal women with (n = 4,340) and without (n = 97,576) incident invasive breast cancer over 10 years post-diagnosis, stratified by 3 age groups (50–59, 60–69, and 70–79).

**Results:**

Postmenopausal women, regardless of breast cancer diagnosis, had similar and high levels of CVD risk factors (e.g., smoking and hypertension) at baseline prior to breast cancer, which were strong predictors of CVD and total mortality over time. CVD affected mostly women age 70–79 with localized breast cancer (79% of breast cancer cases in 70–79 age group): only 17% died from breast cancer and CVD was the leading cause of death (22%) over the average 10 years follow up. Compared to age-matched women without breast cancer, women age 70–79 at diagnosis of localized breast cancer had a similar multivariate-adjusted hazard ratio (HR) of 1.01 (95% confidence interval [CI]: 0.76–1.33) for coronary heart disease, a lower risk of composite CVD (HR = 0.84, 95% CI: 0.70–1.00), and a higher risk of total mortality (HR = 1.20, 95% CI: 1.04–1.39).

**Conclusion:**

CVD was a major contributor to mortality in women with localized breast cancer at age 70–79. Further studies are needed to evaluate both screening and treatment of localized breast cancer tailored to the specific health issues of older women.

## Introduction

With the aging US population and the favorable long term survival following breast cancer [1–4], greater numbers of older breast cancer survivors are at risk for developing aging-related chronic conditions, such as cardiovascular disease (CVD) [5,6]. The median age of diagnosis of incident myocardial infarction (MI) is in the early 70s for women in the US [7]. Patients over age 70 account for about 1/3 of all breast cancer cases [1]. A recent report from the International Society of Geriatric Oncology and the European Society of Breast Cancer Specialists notes that the recommendation for management of breast cancer in older individuals is limited by lack of good evidence and extrapolation of results from younger women with breast cancer [8]. In this paper, outcomes of incident breast cancer among older postmenopausal women were evaluated with emphasis on CVD, the leading cause of morbidity and mortality among older women [9].

CVD and breast cancer share multiple risk factors, such as age, postmenopausal obesity and physical inactivity [10–14]. Many postmenopausal women are already at risk of CVD at their breast cancer diagnosis [15,16]. In addition, successful therapies for breast cancer, such as chemo-, radio- and endocrine therapy, are known to carry short- and long-term risks for CVD [4,17–27]. Some of the more cardiotoxic therapies (e.g., anthracycline-based and targeted chemotherapy) are used primarily for advanced breast cancer (i.e., lymph node-positive regional or distant/metastatic stage) which is more prevalent in younger women. The majority of postmenopausal breast cancer is early stage or localized (i.e., lymph node-negative) at diagnosis, and the extended anti-estrogen therapy (i.e., aromatase inhibitors) is the primary adjuvant therapy, which may pose additional long-term risks of CVD, especially to older postmenopausal patients [17–19,28–30]. The percentage of non-breast cancer deaths, including CVD deaths, among breast cancer survivors increases with advancing age and other comorbidities [6]. However, both physicians and patients are likely to perceive a breast cancer diagnosis as the prime medical priority and overlook CVD risk [19], thereby hindering optimal diagnosis and treatment of comorbidities like CVD risk factors and symptoms particularly in older patients [6,31,32].

Previous studies compared some CVD outcomes over relatively short time period within breast cancer drug trials by intervention arms [30], but age-specific longer-term risks of a wide range of CVD outcomes have not been compared between older women with breast cancer and age-matched women without breast cancer. The prevalence and effect of pre-existing CVD risk factors before diagnosis of breast cancer on CVD after breast cancer has not been studied in longer term longitudinal studies among older breast cancer survivors compared to women without breast cancer. Therefore, we conducted a prospective study within the Women’s Health Initiative (WHI) Extension Study cohort [33] to compare age-specific rates of subsequent composite and individual CVD events (i.e., coronary heart disease [CHD], angina, coronary revascularization, peripheral arterial disease [PAD], and stroke) and mortality outcomes (i.e., total, CVD, and CHD death) between postmenopausal women with and without breast cancer. We also compared prevalence and effects of pre-existing (i.e., baseline) CVD risk factors on CVD outcomes between those with and without breast cancer over 15 years of follow-up from study entry to WHI.

## Materials and methods

Details of the design and conduct of the WHI clinical trials (CTs) and observational study (OS) have been published [33–35]. In brief, the WHI enrolled 161,808 postmenopausal women from 1993 to 1998 at 40 US clinical centers into 4 CTs (two hormone therapy trials and trials of dietary modification and calcium and vitamin D supplementation) (n = 68,132) and an OS (n = 93,676). Postmenopausal status was defined as the absence of menstrual bleeding in women for 1 year, if under age 55, and 6 months, if over age 55. General eligibility required age between 50–79 years, being accessible for follow-up and having predicted survival of greater than 3 years. The CTs had additional eligibility requirements and specific exclusion criteria (e.g., breast cancer at baseline, acute heart attack or stroke in the previous 6 months) [33]. Institutional review board approval was obtained at all WHI clinical centers and at the Clinical Coordinating Center (CCC). All WHI participants at baseline visit provided written informed consent, completed self-administered forms, an interview and a physical examination, and supplied blood samples. Participants in the CTs were followed by annual clinic visits and semiannual questionnaires. Participants in the OS attended a 3-year clinic visit and were followed by annual questionnaires. Follow-up after the original study endpoint in 2005 required re-consent and this was obtained from 76.9% of the participants who then received subsequent follow-up through September 30, 2010. Given that ascertainment protocols in the WHI were modified after 2010, evaluation of events in this study was restricted to women who consented to follow up to 2010 for both breast cancer and comparison women.

### Adjudication process

Incident CVD events and breast cancer cases were initially ascertained by self-report and documented by medical record review. Potential cases were sent to local WHI-physician adjudicators for evaluation and classification. Locally adjudicated cases were then sent to the WHI CCC for central adjudication of selected outcomes [36]. Cases received final adjudication at the CCC with breast cancers centrally coded by trained tumor registry coders using standardized National Cancer Institute Surveillance, Epidemiology, and End Results (SEER) guidelines [37]. Breast cancer hormone receptor status was based on local laboratory assessment. CVD events were defined using prospectively established criteria. Strokes were adjudicated by trained subspecialty physician adjudicators after medical record review.

### Study population

**Fig 1:**
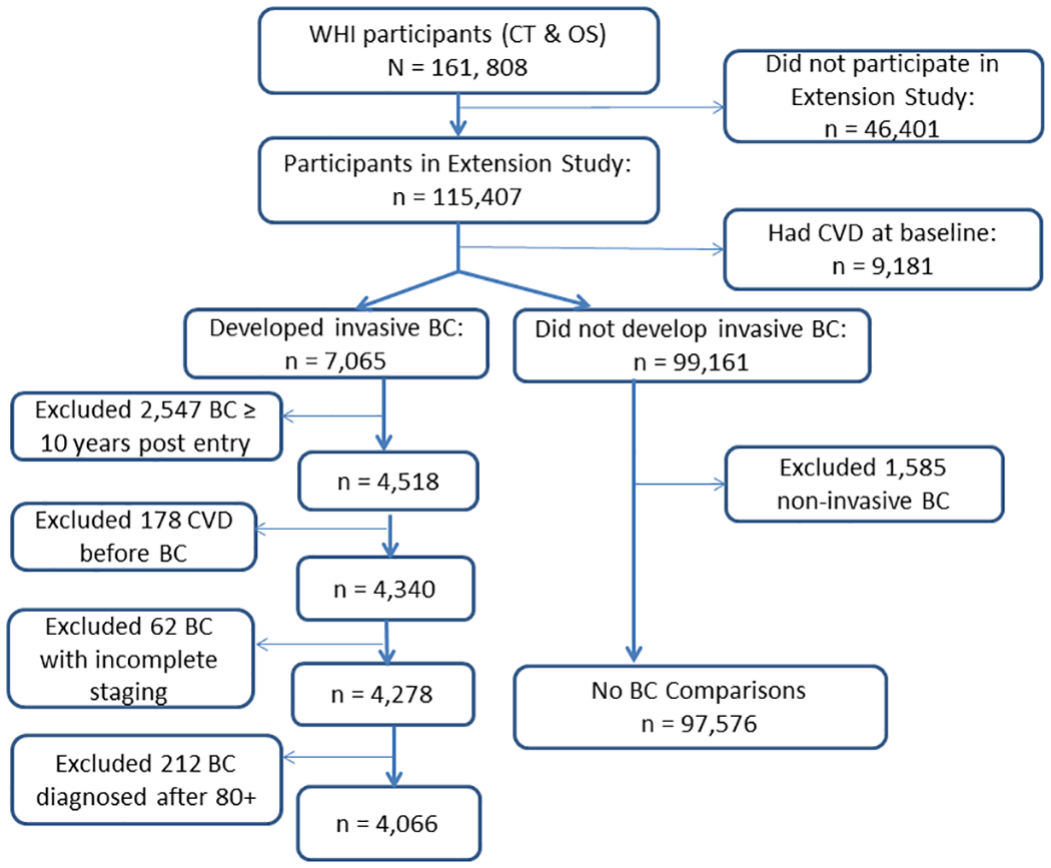
Flow diagram of participants included in the present study. WHI indicates Women’s Health Initiative; CT, clinical trials; OS, observational study; CVD, cardiovascular disease; and BC, breast cancer.

Of 115,407 WHI Extension Study participants, we excluded 9,181 with CVD history at baseline. Of the remaining 106,226 participants, 7,065 developed invasive breast cancer. Of these, only breast cancer cases that occurred within the first 10 years of follow up in the WHI were included because there was very little follow up time for CVD outcomes after diagnosis of breast cancer beyond 10 years from baseline. There were 4,518 incident invasive breast cancer cases. After excluding 178 with incident CVD prior to breast cancer diagnosis, 4,340 invasive breast cancer cases were available for the primary analyses. Of the 99,161 participants with no invasive breast cancer, 1,585 with incident non-invasive breast cancer (i.e., ductal carcinoma in situ or lobular carcinoma in situ) were excluded, resulting in 97,576 comparisons (Fig 1).

Breast cancer stages were classified as localized or not localized based on the absence or presence of lymph node metastasis at the time of diagnosis. There were 466 localized breast cancer (i.e., no lymph node metastasis) cases diagnosed at age 50–59, 1,479 at age 60–69, and 1,164 at age 70–79. Breast cancer cases were also further classified by grade from well differentiated to anaplastic, presence of estrogen or progesterone receptors, and expression of human epidermal growth factor receptor 2 (HER2). The number of women in the WHI Hormone Trials in this study was too small for further separate analysis and included in the overall results, noting no effect on the overall results of the study [38,39].

### Study outcomes

The primary study outcomes were incident CVD events and mortality outcomes directly attributed to CVD and CHD, and all-causes. Composite CVD was defined as the first occurrence of any events of CHD, angina, coronary revascularization, heart failure, PAD, and stroke. CHD was defined as acute MI that required overnight hospitalization or coronary death. The diagnosis of nonfatal MI was confirmed if data in the hospital record met standardized criteria of diagnostic electrocardiographic changes, elevated cardiac-enzyme levels, or both. The presence of angina was confirmed by hospitalization and confirmatory evidence on angiography, diagnostic stress test, or diagnosis by a physician and medical treatment. Coronary revascularization was confirmed by documentation of coronary artery bypass graft, coronary angioplasty, stent, or artherectomy procedures in the medical record. Stroke was confirmed by documentation in the medical record of the rapid onset of a neurologic deficit consistent with stroke and lasting at least 24 hours or until death. The presence of heart failure was confirmed by hospitalization and diagnostic confirmatory tests. We also used the end points of CHD, angina, coronary revascularization, PAD, and stroke as individual outcomes [36,40].

Deaths were categorized as total death, CVD death, and CHD death and were determined by trained physician adjudicators at the CCC based on medical record or death certificate review. CVD mortality included deaths from CHD, cerebrovascular disease, pulmonary embolism, heart failure, and other cardiovascular causes. CHD mortality was defined as death consistent with CHD as the underlying cause plus one or more of the following: hospitalization for MI within 28 days before death, previous angina or MI, death due to a procedure related to CHD or a death certificate consistent with the underlying cause as atherosclerotic CHD [36,40].

### Baseline characteristics and CVD risk factors

Baseline characteristics were obtained from self-administered questionnaires on demographics, health behaviors, medical history, and physical examination [35]. Race/ethnicity was categorized by self-report. Current or past menopausal hormone therapy use was based on interview and review of medication containers. Women randomized to the active drug group in the two hormone therapy trials were considered current users of estrogen alone or estrogen plus progestin, respectively. Current smoking status was determined by self-report. Body mass index (BMI) was calculated as weight (in kilograms) divided by height (in meters) squared (kg/m^2^) based on baseline physical assessment. Waist circumference (cm) was measured after normal expiration over nonbinding undergarments. Blood pressure was measured with standard protocols. Hypertension was defined as either a blood pressure of at least 140/90 mm Hg or hypertension medication use and categorized as *not hypertensive*, *untreated hypertensive*, or *treated hypertensive*. Diabetes status was determined by self-report of treated diabetes or high blood sugar when not pregnant. Hypercholesterolemia was determined by history of cholesterol medication use. Physical activity was defined as a total metabolic equivalent score (MET-hours/week) [33].

### Statistical analyses

Categorical variables were presented as frequency and percentage, and continuous variables were presented as mean ± standard deviation (SD) or median and inter-quartiles, if the distribution was skewed. Chi-square tests were conducted to compare categorical variables. Age-specific incidence or mortality rates per 1,000 person-years and their 95% confidence intervals (CIs) were calculated. Cox models were conducted to estimate hazard ratios (HRs) and 95% CIs for assessing the association of breast cancer status and CVD risk factors with outcomes. The starting points for time-to-event analyses were the WHI study entry for women with and without breast cancer as well as the date of invasive breast cancer for women with breast cancer. The ending point for all participants was the date of the initial CVD event or the end of follow up. Participants were censored at the time of death or the date of last information available. All analyses were 2-sided at alpha = 0.05 and performed with SAS version 9.3 (SAS Institute, Cary, NC).

**Fig 2:**
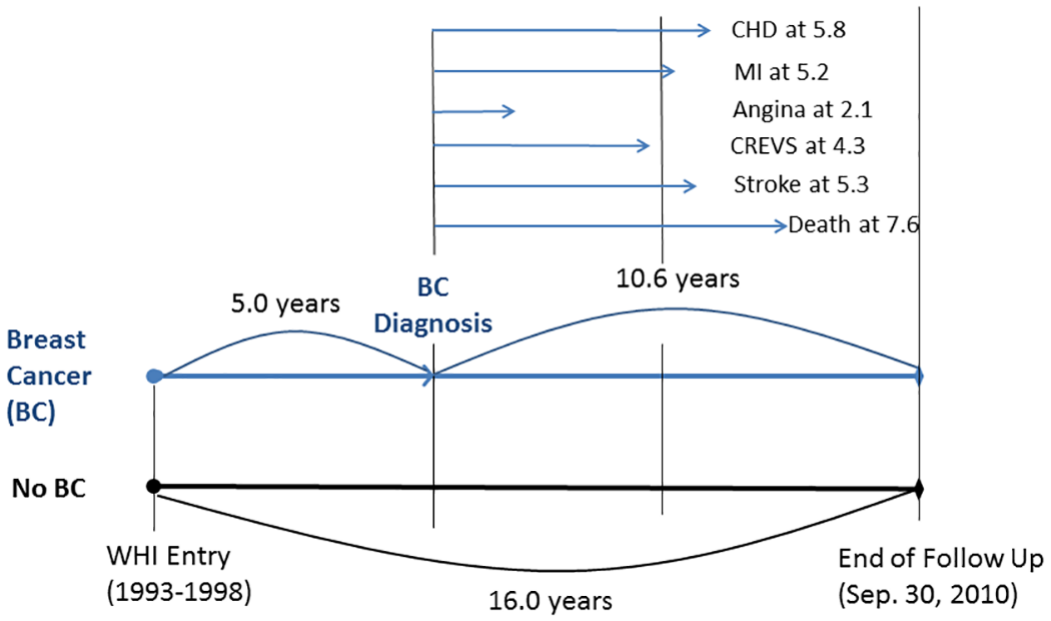
Median years of follow up scheme of the study for women with and without breast cancer. CHD indicates coronary heart disease; MI, myocardial infarction; CREVS, coronary revascularization; and WHI, Women’s Health Initiative.

For women with breast cancer, the analyses were based on age at breast cancer diagnosis and person-years follow up limited to time after the breast cancer diagnosis. To handle the average difference of 5 years between age at entry to WHI and age at diagnosis of breast cancer in breast cancer cases (Fig 2 and S1 Table), several approaches have been used to evaluate the relationship of breast cancer to incidence of CVD and death after diagnosis of breast cancer compared to women who did not develop breast cancer. First, we only included women whose age at the diagnosis of breast cancer and the age at entry to WHI were in the same age categories of 50–59, 60–69, and 70–79. Among women age 70–79 at baseline (n = 838), 220 (26%) breast cancers diagnosed at 80+ years of age were excluded from the analyses (S1 Table). The age-specific rates of subsequent CVD morbidity and mortality outcomes were then compared with women who did not develop breast cancer (i.e., comparisons) using their age at baseline. This resulted in the similar baseline ages of both groups. However, length of follow up time was longer for the comparisons whose follow up time was based on entry to WHI. The mean duration of follow up was 15.7 (SD = 2.0) years for no breast cancer comparisons vs. 10.4 (SD = 3.5) years for breast cancer cases (Fig 2).

The second approach was to adjust for both age at diagnosis and length of follow up. Women with breast cancer were categorized into 3 age groups based on the age at diagnosis of breast cancer but not the age at study entry, and follow up began at the time of breast cancer diagnosis. For the no breast cancer comparisons, length of follow up and baseline age began approximately 5 years after entry to WHI, and all CVD and mortality events that occurred during the first 5 years were excluded. Based on this approach, the age of women at diagnosis of breast cancer and the age at baseline of women in the comparisons were similar. Also, the length of follow up for both breast cancer cases and comparisons became the same. Furthermore, direct age adjustment within the 10-year age groups was done for both groups to further control for any age differences between the groups. These 2 approaches together controlled for both age and length of follow up to avoid potential survivor bias.

## Results

### Characteristics of study population

Of a total of 4,518 incident invasive breast cancer cases, 4,340 had no history of CVD prior to their breast cancer. After excluding breast cancer cases after age 80+, 4,066 cases were available with detailed staging data (Fig 1), including 627 (15%) in age 50–59, 1957 (48%) in age 60–69, and 1482 (36%) in age 70–79 at diagnosis of breast cancer. The average age at breast cancer diagnosis was 68 years (SD = 7.2, median = 67.9). The percentage of localized breast cancer increased by advancing age: 71% at age 50–59 to 76% at age 60–69 to 79% at age 70–79.

The mean time to breast cancer diagnosis for the 4,340 women without CVD at baseline and prior to breast cancer was 5.0 ± 2.8 years (Fig 2). There were 735 deaths among breast cancer cases, and mean and median years to death from breast cancer were 7.5 ± 3.7 and 7.6 years, respectively. For localized breast cancer cases only, mean time to diagnosis was also 5.0 ± 2.7 years (median = 4.9) and 459 deaths occurred at average 8.1 ± 3.4 years (median = 8.1) after localized breast diagnosis.

The CVD risk factors were measured only at baseline entry to WHI. The relationships of risk factors to breast cancer cases with and without subsequent CVD were analyzed in several ways. First, we compared risk factors for CVD that occurred prior to incident breast cancer vs. for CVD events that occurred after breast cancer diagnosis (Table 1). Among breast cancer cases, women who developed CVD since the WHI entry were significantly more likely to be older, have lower levels of education and income, be a current smoker, have a history of hypertension, diabetes, or hypercholesterolemia, have a high waist circumference and be a non-user of hormone therapy at entry to WHI compared to breast cancer cases without follow-up CVD (Table 1). There were few differences in baseline risk factors between women with incident CVD before or after the onset of incident breast cancer (Table 1). Only diabetes and cigarette smoking were significantly different: Having diabetes was associated with CVD before breast cancer; and current and past smoking was associated with CVD after breast cancer. We also compared CVD risk factors at entry to WHI between women with breast cancer and comparisons without breast cancer (S2 Table). There were also few differences in the distribution of risk factors between women with and without breast cancer (Table 1 and S2 Table). Results were similar whether we included all incident CVD (i.e., before or after breast cancer) or just incident CVD after breast cancer diagnosis (Table 1). The characteristics of breast cancer cases (e.g., stage, grade, etc.) were also very similar for women who did or did not develop incident CVD (S3 Table).

**Table 1:** Study entry characteristics and cardiovascular disease (CVD) risk factors by CVD status in all women with incident invasive breast cancer (N = 4,518).

	No CVD since study entry (n = 3,981)	CVD before breast cancer (n = 178)	CVD after breast cancer (n = 359)	All CVD since study entry (n = 537)	[1] vs. [3]	[2] vs. [3]	[1] vs. [4]
	[1] n (%)	[2] n (%)	[3] n (%)	[4] n (%)	p	p	p
**Age at Study Entry**					< .0001	0.936	< .0001
50–59 years	1,350 (33.9)	32 (18.0)	61 (17.0)	93 (17.3)			
60–69 years	1,908 (47.9)	88 (49.4)	183 (51.0)	271 (50.5)			
70–79 years	723 (18.2)	58 (32.6)	115 (32.0)	173 (32.2)			
**Race/Ethnicity**					0.395	0.729	0.293
White	3,546 (89.3)	163 (91.6)	320 (89.1)	483 (89.9)			
Black	215 (5.4)	10 (5.6)	26 (7.2)	36 (6.7)			
Hispanic	84 (2.1)	3 (1.7)	6 (1.7)	9 (1.7)			
Other	127 (3.2)	2 (1.1)	7 (2.0)	9 (1.7)			
**Education**					0.012	0.129	< .0001
≤ High school	625 (15.8)	38 (21.3)	67 (18.7)	105 (19.6)			
Some college	1,358 (34.4)	82 (46.1)	142 (39.7)	224 (41.8)			
≥ College	1,967 (49.8)	58 (32.6)	149 (41.6)	207 (38.6)			
**Study Arm**					0.168	0.787	0.067
Clinical Trial	1,702 (42.8)	85 (47.7)	167 (46.5)	252 (46.9)			
Observational	2,279 (57.3)	93 (52.3)	192 (53.5)	285 (53.1)			
**Body Mass Index (kg/m**^**2**^**)**					0.205	0.171	0.041
< 25	1,427 (36.1)	44 (24.7)	119 (33.2)	163 (30.3)			
25.0–29.9	1,350 (34.2)	68 (38.2)	124 (34.5)	192 (35.7)			
30.0–34.9	729 (18.5)	38 (21.3)	73 (20.3)	111 (20.7)			
35–39.9	299 (7.6)	19 (10.7)	35 (9.8)	54 (10.1)			
≥ 40	141 (3.6)	9 (5.1)	8 (2.2)	17 (3.2)			
**Waist Circumference**					0.010	0.190	< .0001
≤ 88 cm	2,453 (61.8)	87 (48.9)	197 (54.9)	284 (52.9)			
> 88 cm	1,518 (38.2)	91 (51.1)	162 (45.1)	253 (47.1)			
**Smoking**					0.010	0.031	0.049
Never	1,954 (49.7)	98 (55.7)	156 (43.6)	254 (47.6)			
Past	1,757 (44.7)	66 (37.5)	170 (47.5)	236 (44.2)			
Current	220 (5.6)	12 (6.8)	32 (8.9)	44 (8.2)			
**Hypertension**					< .0001	0.177	< .0001
No	2,821 (71.5)	95 (53.4)	213 (59.5)	308 (57.5)			
Yes	1,126 (28.5)	83 (46.6)	145 (40.5)	228 (42.5)			
**Diabetes**					0.002	0.007	< .0001
No	3,835 (96.4)	152 (85.9)	334 (93.0)	486 (90.7)			
Yes	145 (3.6)	25 (14.1)	25 (7.0)	50 (9.3)			
**Hypercholesterolemia**					0.001	0.923	< .0001
No	3,348 (88.8)	140 (82.3)	282 (82.7)	422 (82.6)			
Yes	422 (11.2)	30 (17.7)	59 (17.3)	89 (17.4)			
**Physical Activity (total MET-hours/week)**					0.249	0.833	0.134
< 2.5	877 (23.0)	44 (25.3)	92 (26.6)	136 (26.2)			
2.5–18.24	1,973 (51.8)	94 (54.0)	177 (51.2)	271 (52.1)			
≥ 18.25	956 (25.1)	36 (20.7)	77 (22.2)	113 (21.7)			
**Menopausal Hormone Therapy**					0.004	0.838	0.002
Never	1,478 (37.2)	76 (42.7)	163 (45.4)	239 (44.5)			
Past	543 (13.7)	26 (14.6)	50 (13.9)	76 (14.2)			
Current	1,956 (49.2)	76 (42.7)	146 (40.7)	222 (41.3)			

MET indicates metabolic equivalent score.

### Causes of death by age at diagnosis of breast cancer

Among 627 women age 50–59 at diagnosis of breast cancer, 63 (10%) had died, including 35 of 446 (8%) with localized disease. Breast cancer was the leading cause of death accounting for 36 (57%) of the 63 deaths and CVD for 7 (11%) of the deaths in the group of age 50–59 at the time of breast cancer diagnosis (S4 Table). There were 227 deaths (11.6%) among 1,957 women age 60–69 at the time of diagnosis of breast cancer, including 9% (136 deaths) of 1,479 women with localized disease. Breast cancer accounted for 41% of the deaths and CVD for 14 (6.2%) of the deaths in the women age 60–69 at breast cancer diagnosis (S4 Table).

The situation for women who developed breast cancer at age 70–79 was somewhat different in that 328 (22%) had died and 86 of 328 deaths (26%) were due to breast cancer and 63 (19%) to CVD (Table 2). As noted, 1,164 (79%) of the 1,482 breast cancer cases were localized at the time of diagnosis and 226 (19%) of the women with localized breast cancer had died. Only 17% of the deaths for women with localized disease were due to breast cancer and 49 (22%) deaths from CVD. Breast cancer deaths in this older age group occurred in 39 (3%) of the 1164 women with localized disease over the average 10.6 years of follow up (Table 2 and Fig 2). The percentage of deaths was very high for older individuals age 70+ with either regional or distant disease and about a half of the deaths were due to breast cancer. No other causes of death were substantially different among breast cancer cases vs. no breast cancer comparisons (S5 Table).

**Table 2:** Survival status and cause of death in women with breast cancer at age 70–79.

	Invasive Breast Cancer Diagnosis at Age 70–79			
	**Localized**	**Regional**	**Distant**	**All**
**Survival status**	**n (%)**	**n (%)**	**n (%)**	**n (%)**
**Alive**	938 (80.6)	214 (68.6)	2 (33.3)	1,154 (77.9)
**Dead**	226 (19.4)	98 (31.4)	4 (66.7)	328 (22.1)
**Total (%)**	1,164 (78.5)	312 (21.1)	6 (0.4)	1,482 (100)
	**Localized**	**Regional**	**Distant**	**All**
**Causes of death**	**n (%)**	**n (%)**	**n (%)**	**n (%)**
**Breast Cancer**	39 (17.3)	43 (43.9)	4 (100)	86 (26.2)
**Other Major Cancers** ^a^	14 (6.2)	8 (8.2)	0 (0.0)	22 (6.7)
**Other Cancer Death**	26 (11.5)	5 (5.1)	0 (0.0)	31 (9.5)
**Total CVD**	49 (21.7)	14 (14.3)	0 (0.0)	63 (19.2)
Coronary heart disease	21 (9.3)	9 (9.2)	0 (0.0)	30 (9.1)
Stroke	9 (4.0)	2 (2.0)	0 (0.0)	11 (3.4)
Other CVD	19 (8.4)	3 (3.1)	0 (0.0)	22 (6.7)
**Others** ^b^	98 (43.4)	28 (28.6)	0 (0.0)	126 (38.4)

CVD indicates cardiovascular disease.

^a^ Major cancers include lung, ovarian, and colon cancers.

^b^ Other causes of death include COPD, pneumonia, sepsis, accident, Alzheimer’s disease, etc. For more details, see S5 Table.

### Age-specific risks of CVD and mortality outcomes

Table 3 presents the results for breast cancer cases restricted to the same 10-year age categories by both ages at baseline and breast cancer diagnosis. Total mortality was higher for women with breast cancer than no breast cancer comparisons in all 3 age groups (Table 3). The CI limits of the rates for CVD outcomes, however, were overlapped in all 3 age groups between breast cancer cases and comparisons. CVD incidence was higher for no breast cancer comparisons than for women with breast cancer due to primarily lower rates of coronary revascularizations and hospitalized angina (Table 3). The results were similar with the inclusion of breast cancer cases diagnosed after age 80 (data not shown).

**Table 3:** Cardiovascular disease (CVD) and death in women with localized breast cancer ^a^ vs. women with no breast cancer^a^.

	No Breast Cancer			Localized Invasive Breast Cancer		
	By Age at Baseline (BL)			By Age at BL & Diagnosis ^b^		
	BL Age-adjusted Rate ^c^ per 1,000 Person-Years (95% CI)			BL Age-adjusted Rate ^c^ per 1,000 Person-Years (95% CI)		
	50–59	60–69	70–79	50–59	60–69	70–79 ^d^
**Mean and Median Ages at Study Entry, years**	55, 55	64, 64	73, 72	54, 54	63, 63	72, 72
**CVD** ^e^	3.94 (3.68–4.23)	9.40 (9.02–9.80)	17.75 (16.86–18.68)	3.10 (1.49–6.83)	6.43 (4.29–9.64)	12.41 (8.46–18.23)
**Coronary Heart Disease (CHD)**	1.14 (1.00–1.29)	2.67 (2.47–2.88)	5.56 (5.09–6.07)	0.53 (0.13–2.10)	1.65 (0.77–3.88)	5.33 (2.83–10.20)
**MI**	0.97 (0.84–1.11)	2.18 (2.00–2.37)	4.09 (3.69–4.54)	0.38 (0.08–1.97)	1.21 (0.54–3.30)	3.78 (1.84–8.15)
**Angina**	0.73 (0.62–0.86)	1.61 (1.46–1.78)	2.40 (2.10–2.75)	0.97 (0.23–4.55)	1.43 (0.58–3.59)	1.15 (0.30–4.72)
**Coronary Revascularization**	1.63 (1.47–1.81)	3.70 (3.46–3.95)	4.97 (4.53–5.46)	1.43 (0.51–5.17)	3.30 (1.80–6.05)	3.30 (1.51–7.54)
**PAD**	0.19 (0.14–0.26)	0.51 (0.43–0.61)	0.79 (0.63–1.00)	0.41 (0.09–1.99)	0.31 (0.04–2.20)	0.43 (0.09–2.11)
**Stroke**	0.78 (0.67–0.91)	2.18 (2.01–2.37)	4.69 (4.26–5.16)	0.29 (0.07–1.18)	2.37 (1.17–4.83)	5.23 (2.73–10.11)
**Total Death**	2.18 (1.99–2.39)	5.96 (5.67–6.27)	15.50 (14.72–16.33)	6.25 (3.46–11.33)	8.90 (6.22–12.80)	21.95 (15.97–30.17)
**CVD Death**	0.39 (0.32–0.49)	1.39 (1.26–1.55)	5.00 (4.57–5.48)	0.67 (0.20–2.28)	0.81 (0.25–2.76)	6.20 (3.43–11.29)
**CHD Death**	0.19 (0.14–0.25)	0.60 (0.51–0.70)	1.98 (1.71–2.29)	0.15 (0.02–1.04)	0.43 (0.10–1.95)	1.96 (0.71–5.71)
**Breast Cancer Death**	NA	NA	NA	2.34 (0.92–6.51)	2.24 (1.09–4.64)	3.13 (1.37–7.27)

CI indicates confidence interval; MI, myocardial infarction; PAD, peripheral arterial disease; and NA, not applicable.

^a^ Excluding: 1) women had CVD at baseline; 2) women had CVD prior to breast cancer; 3) women with non-invasive breast cancer; 4) time to breast cancer ≥ 10 years; and 5) breast cancer diagnosed after age 80.

^b^ Analyses was restricted to women whose age at entry and age at diagnosis of breast cancer are in the same age group and excluded women with age at diagnosis of 80+.

^c^ The baseline age adjustment was done by the rate at each age group that was calculated from the combined groups of no breast cancer comparisons and breast cancer cases.

^d^ There was a total of 484 localized breast cancer cases at age 70–79 with mean age 76 ± 2.5 years (median = 76) at breast cancer.

^e^ CVD events include CVD deaths.

The number of CVD events was very low among breast cancer cases under the age of 70 regardless of stages of breast cancer at diagnosis (S6 Table). There were, for example, only 7 CHD events at age 50–59 and 39 at age 60–69. Therefore, we further focused on women with localized disease at age 70–79. In the analysis in Table 4, the first 5 years of follow up, as noted, for comparisons was eliminated. The age was reset and follow up years began at that point. The age and length of person-years follow up were similar for comparisons and breast cancer cases within the 70–79 age group (Table 4). Women age 70–79 with localized breast cancer had: (1) a lower multivariate-adjusted risk of composite CVD (HR = 0.84, 95% CI: 0.70–1.00); (2) similar risks of CHD and MI; (3) a lower risk of coronary revascularization (HR = 0.72, 95% CI: 0.52–0.99); (4) a similar risk of stroke; and (5) a slightly higher risk of total mortality (HR = 1.20, 95% CI: 1.04–1.39), but no differences in CVD and CHD mortality outcomes (Table 4).

**Table 4:** Localized breast cancer status and cardiovascular disease (CVD) and mortality outcomes in the 70–79 age group^a^.

Event	Localized Breast Cancer	Total # per Group	# of Event	Mean person-years	Age ^b^-adjusted Rate per 1,000 Person-Years (95% CI)	Age ^b^-adjusted HR (95% CI)	Multivariate ^c^-adjusted HR (95% CI)
**CVD** ^d^	**No** ^e^	31,833	4,620	9.57	14.72 (14.00, 15.48)	1.00	1.00
	**Yes** ^f^	1,164	140	9.20	11.96 (8.98, 15.92)	0.83 (0.70, 0.98)	0.84 (0.70, 1.00)
**Coronary Heart Disease (CHD)**	**No**	33,379	1,694	10.04	4.86 (4.48, 5.29)	1.00	1.00
	**Yes**	1,164	55	9.49	4.56 (2.89, 7.19)	0.98 (0.75, 1.28)	1.01 (0.76, 1.33)
**Myocardial Infarction**	**No**	33,385	1,239	10.05	3.58 (3.24, 3.94)	1.00	1.00
	**Yes**	1,164	37	9.49	3.06 (1.77, 5.33)	0.90 (0.65, 1.24)	0.92 (0.66, 1.29)
**Angina**	**No**	33,136	409	10.12	1.21 (1.02, 1.43)	1.00	1.00
	**Yes**	1,164	7	9.58	0.57 (0.16, 2.07)	0.49 (0.23, 1.03)	0.47 (0.21, 1.05)
**Coronary Revascularization**	**No**	33,101	1,705	9.92	5.14 (4.74, 5.59)	1.00	1.00
	**Yes**	1,164	39	9.45	3.19 (1.86, 5.50)	0.68 (0.50, 0.94)	0.72 (0.52, 0.99)
**Peripheral Arterial Disease**	**No**	33,717	315	10.18	0.89 (0.74, 1.09)	1.00	1.00
	**Yes**	1,164	5	9.62	0.40 (0.09, 1.88)	0.49 (0.20, 1.19)	0.43 (0.16, 1.16)
**Stroke**	**No**	33,490	1,458	10.06	4.17 (3.81, 4.56)	1.00	1.00
	**Yes**	1,164	46	9.50	3.85 (2.36, 6.33)	0.94 (0.70, 1.26)	1.02 (0.75, 1.37)
**Total Death**	**No**	33,790	5,196	10.22	14.32 (13.66, 15.02)	1.00	1.00
	**Yes**	1,164	226	9.64	18.48 (14.75, 23.16)	1.24 (1.08, 1.42)	1.20 (1.04, 1.39)
**CVD Death**	**No**	33,790	1,443	10.22	3.93 (3.59, 4.29)	1.00	1.00
	**Yes**	1,164	49	9.64	4.01 (2.48, 6.50)	0.99 (0.74, 1.32)	0.92 (0.67, 1.26)
**CHD Death**	**No**	33,790	606	10.22	1.64 (1.43, 1.89)	1.00	1.00
	**Yes**	1,164	21	9.64	1.73 (0.84, 3.61)	1.02 (0.66, 1.60)	1.03 (0.65, 1.65)
**BC Death**	**Yes**	1,164	39	9.64	3.16 (1.84, 5.43)	NA	NA

CI indicates confidence interval; HR, hazard ratio; BC, breast cancer; and NA, not applicable.

^a^ Excluding: 1) women had CVD at baseline; 2) women had CVD prior to breast cancer; 3) women with non-invasive breast cancer; 4) time to breast cancer ≥ 10 years; and 5) breast cancer diagnosed after age 80.

^b^ Adjusted for age at baseline (BL) for no breast cancer comparisons and age at breast cancer for women with breast cancer.

^c^ Cox models were adjusted for following covariates: age, ethnicity, education, body mass index, waist circumference, smoking, hypertension, diabetes, and hypercholesterolemia.

^d^ CVD events include CVD deaths.

^e^ Among no breast cancer women at BL age 70–79, the first 5 years of follow up were excluded and the BL age was then re-determined. Mean and median ages at BL for no breast cancer women were 73.7 ± 2.7 and 73.0 years of age, respectively.

^f^ Breast cancer diagnoses after age 80 were exclude. For women with localized breast cancer at 70–79, mean and median ages at breast cancer were 74.3 ± 2.7 and 74.0 years of age, respectively.

**Fig 3:**
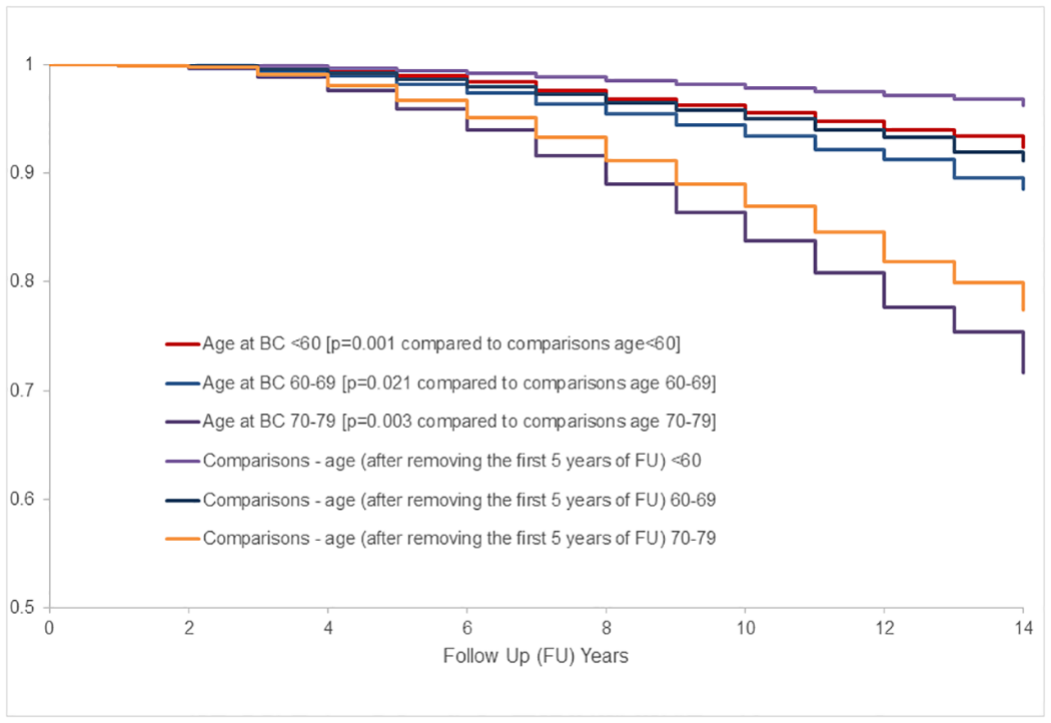
Age adjusted survival curves for total mortality among women with localized breast cancer vs. no breast cancer comparisons. BC indicates breast cancer. For more details regarding each age group, see S1–S3 Figs.

Kaplan Meier survival analysis using analytical approaches similar to Table 4 (e.g., excluding the first 5 years of follow up for comparisons) showed better overall survival for no breast cancer comparisons than localized breast cancer cases within each age group (Fig 3). For the 70–79 age group, estimated lifespan table showed that survivorship after 10 years follow up was 85.0% (95% CI: 84.6–85.4) for no breast cancer comparisons (4,595 deaths out of 33,790 comparisons) and 80.8% (95% CI: 78.0–83.2) for breast cancer cases (183 deaths among 1,164 women with localized breast cancer). There were only 17 deaths among 446 women with localized breast cancer age 50–59 and estimated 10 year survival of 96% (95% CI: 94–98) as compared to 98% (95% CI: 97–99) estimated survival for 14,473 no breast cancer women; and in 60–69 age group, the 10 year survival of 92% (95% CI: 91–94) for 1,479 localized breast cancer as compared to 94% (95% CI: 94–97) for 44,871 comparisons (Fig 3).

A supplementary analysis was conducted to further compare events rates using age at entry rather than age at diagnosis of breast cancer for both localized breast cancer and all invasive breast cancer stratified by ages 50–59, 60–69, and 70–79 (S7 Table). Person-years follow up was restricted to time after breast cancer diagnosis. Total mortality rates were highest for all invasive breast cancer then localized and no breast cancer (S7 Table). There was little difference in CVD, CHD and mortality rates between invasive and localized breast cancer. CVD and CHD rates were also similar for women without breast cancer.

Because mortality follow up was available for the total sample of WHI, we conducted a sensitivity analysis for the mortality results in Table 3 including all WHI participants rather than the 76.9% of the total WHI participants that continued in the follow up after 2005 to 2010. The total mortality rate for participants age 70–79 with no breast cancer was 14.6 (95% CI: 14.1–15.0) and for breast cancer cases, 21.9 (95% CI: 18.3–26.3) per 1,000 person-years, practically the same as for the limited sample used for this study. Results were also similar in the 50–59 and 60–69 age groups and for specific causes of death and CVD outcomes.

### Pre-diagnosis risk factors and post-diagnosis CVD outcomes

Table 5 showed similar associations between the seven traditional CVD risk factors (smoking, hypertension, diabetes, hypercholesterolemia, BMI, waist circumference, and physical activity) and CHD incidence between women who developed incident CHD before and after breast cancer diagnosis. Age-adjusted HRs for CHD by risk factors were also similar between women with and without breast cancer (S8 Table). About 41% of each group of women with and without breast cancer had at least one of 4 major CVD risk factors (i.e., smoking, hypertension, diabetes, and hypercholesterolemia).

**Table 5:** Associations between baseline risk factors and coronary heart disease (CHD) in women with breast cancer.

	CHD incidence since WHI study entry (N = 4,518)				CHD incidence after breast cancer diagnosis ^a^ (N = 4,340)			
Baseline Risk Factors	Total N	CHD n (%)	Age-adjusted Rate per 1,000 Person-years (95% CI)	Age-adjusted HR (95% CI)	Total N	CHD n (%)	Age-adjusted Rate per 1,000 Person-years (95% CI)	Age-adjusted HR (95% CI)
	N	n (%)						
**Current Smoking**								
No	4,201	174 (4.14)	2.58 (2.01, 3.32)	1.00	4,037	124 (3.07)	2.78 (2.08, 3.75)	1.00
Yes	264	20 (7.58)	6.15 (2.99, 12.74)	2.40 (1.50, 3.82)	252	17 (6.75)	8.74 (4.05, 19.05)	3.12 (1.87, 5.20)
**Hypertension**								
No	3,129	104 (3.32)	2.17 (1.58, 3.01)	1.00	2,901	78 (2.57)	2.47 (1.72, 3.58)	1.00
Yes	1,354	91 (6.72)	3.97 (2.79, 5.74)	1.79 (1.35, 2.37)	1,229	64 (5.04)	4.27 (2.81, 6.61)	1.67 (1.19, 2.32)
**Diabetes**								
No	4,321	176 (4.07)	2.57 (2.01, 3.30)	1.0	4,169	134 (3.21)	2.97 (2.25, 3.94)	1.0
Yes	195	19 (9.74)	6.24 (2.86, 14.17)	2.26 (1.41, 3.64)	170	8 (4.71)	4.51 (1.34, 15.79)	1.32 (0.65, 2.69)
**Hypercholesterolemia**								
No	3,770	146 (3.87)	2.47 (1.88, 3.25)	1.00	3,630	107 (2.95)	2.75 (2.01, 3.78)	1.00
Yes	511	38 (7.44)	4.52 (2.61, 8.03)	1.78 (1.24, 2.54)	481	28 (5.82)	5.12 (2.73, 10.03)	1.80 (1.19, 2.73)
**Body Mass Index**								
< 25 kg/m^2^	1,590	60 (3.77)	2.32 (1.56, 3.54)	1.00	1,546	49 (3.17)	2.84 (1.86, 4.51)	1.00
25.0–29.9 kg/m^2^	1,542	66 (4.28)	2.64(1.76, 4.00)	1.16 (0.82, 1.65)	1,474	46 (3.12)	2.80 (1.74, 4.61)	1.03 (0.69, 1.54)
30.0–34.9 kg/m^2^	840	35 (4.17)	2.55 (1.47, 4.54)	1.12 (0.74, 1.70)	802	26 (3.24)	2.83 (1.52, 5.53)	1.06 (0.66, 1.70)
35–39.9 kg/m^2^	353	27 (7.65)	6.30 (3.35, 11.87)	2.64 (1.67, 4.16)	334	17 (5.09)	6.55 (2.97, 14.50)	2.21 (1.27, 3.85)
≥ 40 kg/m^2^	158	7 (4.43)	2.79 (0.82, 12.47)	1.47 (0.67, 3.22)	149	4 (2.68)	2.69 (0.48, 16.20)	1.03 (0.37, 2.87)
**Waist Circumference**								
≤ 88 cm	2,737	104 (3.80)	2.39 (1.75, 3.30)	1.00	2,650	82 (3.09)	2.87 (2.05, 4.09)	1.00
> 88 cm	1,771	91 (5.14)	3.22 (2.26, 4.61)	1.39 (1.05, 1.84)	1,680	60 (3.57)	3.25 (2.10, 5.06)	1.18 (0.84, 1.64)
**Physical Activity (total MET-hours/week)**								
< 2.5	1,013	49 (4.84)	3.17 (1.99, 5.11)	1.00	969	34 (3.51)	3.37 (1.95, 5.96)	1.00
2.5–18.24	2,244	99 (4.41)	2.74 (1.97, 3.84)	0.83 (0.59, 1.18)	2,150	71 (3.30)	3.01 (2.05, 4.49)	0.87 (0.58, 1.30)
≥ 18.25	1,069	39 (3.65)	2.30 (1.37, 3.91)	0.67 (0.44, 1.03)	1,033	32 (3.10)	2.84 (1.63, 5.09)	0.79 (0.49, 1.28)

MET indicates metabolic equivalent score.

^a^ Follow up time began at the diagnosis of breast cancer.

**Fig 4:**
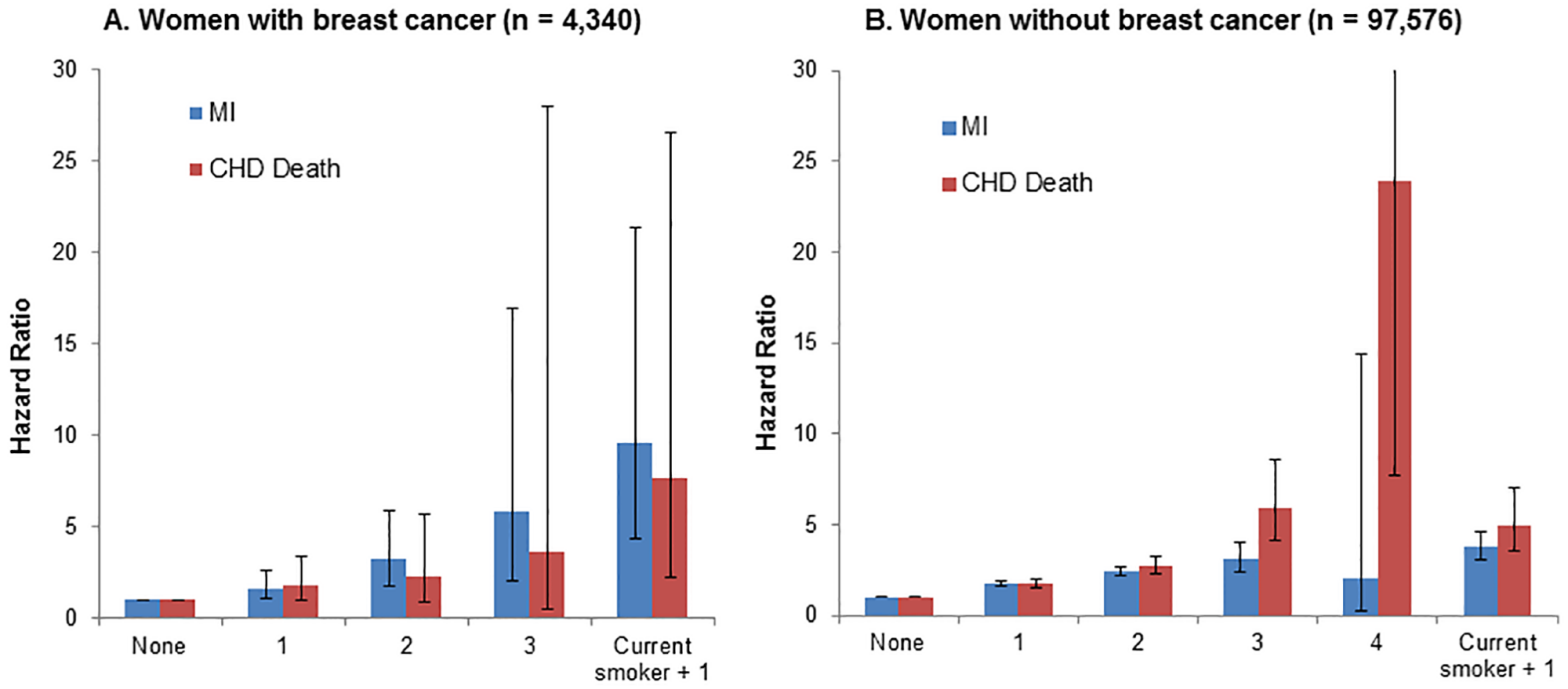
Hazard ratios of myocardial infarction (MI) and coronary heart disease (CHD)-related death by the number of baseline risk factors (i.e., smoking, hypertension, hypercholesterolemia, and diabetes) in women with (A) and without (B) breast cancer ^a^. ^a^Cox models were adjusted for age, ethnicity, education, body mass index, and waist circumference.

In both groups, presence of more baseline CVD risk factors was significantly associated with higher risks of subsequent MI and CHD death at p values ≤ .0001 (Fig 4). Women with breast cancer who reported to be a current smoker with one of the other major risk factors (n = 71, 28% of 252 current smokers) had the greatest risks of MI incidence (HR = 9.61, 95% CI: 4.34–21.32) and CHD death (HR = 7.68, 95% CI: 2.22–26.55), compared to those with no risk factors (Fig 4).

## Discussion

The majority (79%) of older women age 70–79 had localized breast cancer at the time of diagnosis. There was a small but significant increase in total mortality (HR = 1.20) among women age 70–79 who developed localized breast cancer than no breast cancer comparisons. A crude estimate showed about 60 excess deaths including 39 (65%) breast cancer deaths among women with localized breast cancer at age 70–79 than the no breast cancer comparisons. CVD incidence was significantly lower for breast cancer cases than comparisons primarily due to much lower incidence of coronary revascularization, the most common CVD diagnosis among older women. The lower rate of coronary revascularization in older women with localized breast cancer could be due to under-diagnosis of CHD symptoms that may mimic side effects of surgical treatment and radiotherapy for breast cancer [6,15]. Indeed, the standard treatment course for the majority of localized breast cancer cases includes surgery (e.g., lumpectomy, mastectomy) as primary followed by multimodal adjuvant therapies such as radiotherapy and endocrine therapy [1,4]. An ongoing follow up (Life and Longevity After Cancer [LILAC] study) in the WHI is evaluating effects of pharmacological and other therapies for breast cancer on morbidity and mortality with recurrent breast cancer.

CVD was the leading cause of death over 10 years of follow up for women with localized breast cancer diagnosed at 70 years and older based on a strict formal adjudication process of the WHI study by thorough medical record review to determine breast cancer and CVD outcomes. Deaths due to breast cancer in the WHI included only those adjudicated to breast cancer, which provide more accurate data compared to studies that use all deaths after the diagnosis of breast cancer or death certificate classification with possible overestimation of breast cancer deaths [41]. A retrospective cohort study based on the SEER-Medicare database reported that CVD was the primary cause of death followed by breast cancer among older (≥ 66 years) survivors from early stage or localized breast cancer, particularly in those who were ages 75 years and older [6].

Few studies have compared predictive performance of CVD risk factors on CVD outcomes in women with breast cancer [42]. Traditional CVD risk factors (e.g., smoking and history of hypertension and hypercholesterolemia) at baseline years before diagnosis of incident breast cancer were strong predictors of subsequent CVD incidence, regardless of breast cancer status. Women with breast cancer remain at a high risk of CVD as other older women in the US because of their high prevalence of risk factors for CVD.

These new results on the prospective evaluation of CVD risk factors prior to breast cancer extend findings of Haque and colleagues [42] who found post- diagnosis smoking history, diabetes, and hypertension assessed after breast cancer were stronger predictors of CVD than breast cancer status. Timely identification and control of CVD risk factors should be a high priority for older women who develop breast cancer and will likely improve the overall survivorship of older women with localized breast cancer.

This study also provides clinical implications for the screening and management of breast cancer as well as CVD and other comorbidities for older women age 70+ at diagnosis of breast cancer. Mortality remains very high for women with non-localized or advanced (i.e., regional or distant) breast cancer. The goal of mammography is to increase the percentage of early detected breast cancer so that the treatment will effectively reduce mortality from breast cancer. In the WHI, mammographic examinations were required annually for women in the hormone therapy arm of the trial, every other year for those in the dietary modification arm, but were not required for participants in the WHI-OS. There are no clinical guidelines and trials specifically focusing on breast cancer screening among older women [43,44]. An evaluation of time between last mammography screening and breast cancer diagnosis in the WHI among women age 75+ at time of diagnosis suggested a benefit of more recent screening based on reduced deaths due to breast cancer [43] but not reduction in deaths to other causes. There is also little evidence of the benefits of drug therapies or nonpharmacological approaches, such as exercise or nutrition, in reducing total or CVD morbidity and mortality in the 70+ age groups with localized, lymph node-negative breast cancer, a growing phenotype of breast cancer with the aging population [27,29,45]. During the time period of this study (1993–2010), the breast cancer death rates in ages 65–84, have declined in both black and white women [3,46]. It is not known whether the decline is due to better detection and treatment of breast cancer or is consistent with overall better survival and low total mortality rates of older women in the US irrespective of breast cancer diagnosis.

The strengths of this study include the prospective study design, the large, well-characterized diverse WHI population of postmenopausal women with and without breast cancer, breast cancer and CVD outcomes formally adjudicated by medical record review, comprehensive data on CVD risk factors, and a follow-up period of more than a decade for the breast cancer cases. Our study has, however, a few limitations. First, some of the baseline CVD risk factors were based on self-report. However, a validation study revealed high concordance of self-report of diabetes with medical record review [47,48]. Second, we only examined selected CVD risk factors; we did not examine other risk factors such as alcohol consumption, diet, family history of CVD, statin use, or detailed reproductive and hormonal factors. Third, women in the WHI were more likely to have been screened and treated for CVD risk factors than the general population [43]. Lastly, we did not have information on potentially cardiotoxic breast cancer therapies such as chemotherapy or left-sided radiation. Such effects are likely small in women with localized breast cancer. The WHI LILAC study is underway and is designed to obtain these data. A recent report by the Early Breast Cancer Trialists’ Collaborative Group [45] noted a reduction in breast cancer recurrence and mortality rates after breast cancer comparing aromatase inhibitors vs. tamoxifen but did not evaluate CVD events or CVD deaths compared to women without breast cancer, especially in older age group. Evaluation of outcomes in these clinical trials should include age-specific assessments, especially for the 70+ age group.

In conclusion, the majority of older postmenopausal women had localized breast cancer (79%) with good long term survival. Mostly affecting women of age 70+, CVD was the leading cause of death and a major contributor to total mortality in women age 70–79 with localized breast cancer. High prevalence of CVD risk factors prior to breast cancer was associated with CVD and total mortality over time. Optimal efforts to prevent and treat CVD and other diseases after localized breast cancer may have a bigger effect on survival of these older women than treatment that only focus on breast cancer [16,49–51]. A small increase in morbidity or mortality due to adverse effects of anti-cancer therapies may mitigate any breast cancer-specific survival benefit of those therapies for older women with localized breast cancer. Because of the small number of deaths due to localized breast cancer, therapies with broader impact than only against breast cancer may be beneficial in improving morbidity and mortality outcomes for the majority of older women with localized breast cancer.

## Supporting information

S1 Table Age at baseline and age at breast cancer among women with breast cancer. ^a^ n = 212 for age 70–74.9, and n = 406 for age 75–79.9 at breast cancer diagnosis.
(PDF)

S2 Table Study entry characteristics and cardiovascular disease (CVD) risk factors by CVD status in women without incident breast cancer. MET indicates metabolic equivalent score.
(PDF)

S3 Table Breast cancer characteristics by cardiovascular disease (CVD) status in women with invasive breast cancer. HER2 indicates human epidermal growth factor receptor 2. ^a^Chi-square test included missing category. ^b^Chi-square test did not include missing category.
(PDF)

S4 Table Survival status and cause of death in women with invasive breast cancer at ages 50–59 and 60–69. CVD indicates cardiovascular disease.
(PDF)

S5 Table Details of other causes of death in women by age at localized breast cancer diagnosis and women without breast cancer by age at study entry. (PDF)

S6 Table The number of cardiovascular disease (CVD) events and death outcomes by age at breast cancer diagnosis in women with invasive breast cancer (N = 4,340). (PDF)

S7 Table Events rates of cardiovascular disease (CVD) and mortality by baseline (BL) age groups. CHD indicates coronary heart disease; MI, myocardial infarction; REVASC, revascularization; and PAD, peripheral arterial disease.
(PDF)

S8 Table Associations between baseline risk factors and coronary heart disease (CHD) in women without breast cancer. HR indicates hazard ratio; CI, confidential interval; and MET, metabolic equivalent score.
(PDF)

S1 Fig Age adjusted survival curves for total mortality with 95% confidence interval in 70–79 age group: Women with localized breast cancer vs. no breast cancer comparisons. BC indicates breast cancer; and FU, follow up.
(PPTX)

S2 Fig Age adjusted survival curves for total mortality with 95% confidence interval in 60–69 age group: Women with localized breast cancer vs. no breast cancer comparisons. BC indicates breast cancer; and FU, follow up.
(PPTX)

S3 Fig Age adjusted survival curves for total mortality with 95% confidence interval in 50–59 age group: Women with localized breast cancer vs. no breast cancer comparisons. BC indicates breast cancer; and FU, follow up.
(PPTX)

## References

[1] Harbeck N, Gnant M. Breast cancer. Lancet. 2017;389:1134–50. doi: 10.1016/S0140-6736(16)31891-8 27865536 10.1016/S0140-6736(16)31891-8

[2] Ortman JM, Velkoff VA, Hogan H. An aging nation: the older population in the United States. Washington, DC.: U.S. Census Bureau, 2014.

[3] Richardson LC, Henley J, Miller JW, Massetti G, Thomas CC. Patterns and trends in age-specific black-white differences in breast cancer incidence and mortality—United States, 1999–2014. MMWR Morb Mortal Wkly Rep. 2016;65(40). 10.15585/mmwr.mm6540a1 27736827

[4] American Cancer Society. Cancer treatment and survivorship facts & figures 2016–2017. Atlanta: American Cancer Society, 2016.

[5] Colzani E, Liljegren A, Johansson ALV, Adolfsson J, Hellborg H, Hall PFL, et al Prognosis of patients with breast cancer: causes of death and effects of time since diagnosis, age, and tumor characteristics. J Clin Oncol. 2011;29(30):4014–21. doi: 10.1200/JCO.2010.32.6462 21911717 10.1200/JCO.2010.32.6462

[6] Patnaik JL, Byers T, DiGuiseppi C, Dabelea D, Denberg TD. Cardiovascular disease competes with breast cancer as the leading cause of death for older females diagnosed with breast cancer: a retrospective cohort study. Breast Cancer Research. 2011;13(3):R64 doi: 10.1186/bcr2901 21689398 10.1186/bcr2901 PMC3218953

[7] Kuller LH, Lopez OL, Mackey RH, Rosano C, Edmundowicz D, Becker JT, et al Subclinical cardiovascular disease and death, dementia, and coronary heart disease in patients 80+ years. J Am Coll Cardiol. 2016;67(9):1013–22. doi: 10.1016/j.jacc.2015.12.034 26940919 10.1016/j.jacc.2015.12.034 PMC5502352

[8] Biganzoli L, Wildiers H, Oakman C, Marotti L, Loibl S, Kunkler I, et al Management of elderly patients with breast cancer: updated recommendations of the International Society of Geriatric Oncology (SIOG) and European Society of Breast Cancer Specialists (EUSOMA). Lancet Oncol. 2012;13:e148–e160. doi: 10.1016/S1470-2045(11)70383-7 22469125 10.1016/S1470-2045(11)70383-7

[9] Heron M. Deaths: Leading causes for 2014. Natl Vital Stat Rep. 2016;65(5). 27376998

[10] Eliassen AH, Colditz GA, Rosner B, Willett WC, Hankinson SE. Adult weight change and risk of postmenopausal breast cancer. JAMA. 2006;296:193–201. doi: 10.1001/jama.296.2.193 16835425 10.1001/jama.296.2.193

[11] McTigue KM, Chang Y-F, Eaton C, Garcia L, Johnson KC, Lewis CE, et al Severe obesity, heart disease, and death among white, African American, and Hispanic postmenopausal women. Obesity. 2014;22(3):801–10. doi: 10.1002/oby.20224 24493096 10.1002/oby.20224

[12] Nichols HB, Trentham-Dietz A, Egan KM, Titus-Ernstoff L, Holmes MD, Bersch AJ, et al Body mass index before and after breast cancer diagnosis: associations with all-cause, breast cancer, and cardiovascular disease mortality. Cancer Epidemiol Biomarkers Prev. 2009;18(5):1403–9. doi: 10.1158/1055-9965.EPI-08-1094 19366908 10.1158/1055-9965.EPI-08-1094 PMC2715918

[13] Hildebrand JS, Gapstur SM, Campbell PT, Gaudet MM, Patel AV. Recreational physical activity and leisure-time sitting in relation to postmenopausal breast cancer risk. Cancer Epidemiol Biomarkers Prev. 2013;22(10):1906–12. doi: 10.1158/1055-9965.EPI-13-0407 24097200 10.1158/1055-9965.EPI-13-0407

[14] Reddigan JI, Ardern CI, Riddell MC, Kuk JL. Relation of physical activity to cardiovascular disease mortality and the influence of cardiometabolic risk factors. Am J Cardiol. 2011;108:1426–31. doi: 10.1016/j.amjcard.2011.07.005 21855834 10.1016/j.amjcard.2011.07.005

[15] Bardia A, Arieas ET, Zhang Z, DeFilippis A, Tarpinian K, Jeter S, et al Comparison of breast cancer recurrence risk and cardiovascular disease incidence risk among postmenopausal women with breast cancer. Breast Cancer Res Treat. 2012;131:907–14. doi: 10.1007/s10549-011-1843-1 22042368 10.1007/s10549-011-1843-1 PMC3582017

[16] Brarac A, Murtagh G, Carver JR, Chen MH, Freeman AM, Herrmann J, et al Cardiovascular health of patients with cancer and cancer survivors: A roadmap to the next level. J Am Coll Cardiol. 2015;65(25):2739–46. doi: 10.1016/j.jacc.2015.04.059 26112199 10.1016/j.jacc.2015.04.059 PMC4484773

[17] Hozumi Y, Suemasu K, Takei H, Aihara T, Takehara M, Saito T, et al The effect of exemestane, anastrozole, and tamoxifen on lipid profiles in Japanese postmenopausal early breast cancer patients: final results of National Surgical Adjuvant Study BC 04, the TEAM Japan sub-study. Ann Oncol. 2011;22:1777–82. doi: 10.1093/annonc/mdq707 21285133 10.1093/annonc/mdq707

[18] Amir E, Seruga B, Niraula S, Carlsson L, Ocana A. Toxicity of adjuvant endocrine therapy in postmenopausal breast cancer patients: A systematic review and meta-analysis. J Natl Cancer Instit. 2011;103:1–11. 10.1093/jnci/djr242 21743022

[19] Cuppone F, Bria E, Verma S, Pritchard KI, Gandhi S, Milella M, et al Do adjuvant aromatase inhibitors increase the cardiovascular risk in postmenopausal women with early breast cancer?: Meta-analysis of randomized trials. Cancer. 2008;112:260–7. doi: 10.1002/cncr.23171 18041059 10.1002/cncr.23171

[20] Albini A, Pennesi G, Donatelli F, Cammarota R, De Flora S, Noonan DM. Cardiotoxicity of anticancer drugs: The need for cardio-oncology and cardio-oncological prevention. J Natl Cancer Instit. 2010;102:14–25. 10.1093/jnci/djp440 PMC2802286 20007921

[21] Bowles EJA, Wellman R, Feigelson HS, Onitilo AA, Freedman AN, Delate T, et al Risk of heart failure in breast cancer patients after anthracycline and trastuzumab treatment: A retrospective cohort study. J Natl Cancer Instit. 2012;104:1293–305. 10.1093/jnci/djs317 PMC3433392 22949432

[22] Darby SC, Ewertz M, McGale P, Bennet AM, Blom-Goldman U, Bronnum D, et al Risk of ischemic heart disease in women after radiotherapy for breast cancer. N Engl J Med. 2013;368(11):987–98. doi: 10.1056/NEJMoa1209825 23484825 10.1056/NEJMoa1209825

[23] Du X, Xia R, Liu C-C, Cormier JN, Xing Y, Hardy D, et al Cardiac toxicity associated with anthracycline-containing chemotherapy in older women with breast cancer. Cancer. 2009;115:5296–308. doi: 10.1002/cncr.24621 19672997 10.1002/cncr.24621

[24] Hooning MJ, Botma A, Aleman BMP, Baaijens MHA, Bartelink H, Klijn JGM, et al Long-term risk of cardiovascular disease in 10-year survivors of breast cancer. J Natl Cancer Instit. 2007;99:365–75. 10.1093/jnci/djk064 17341728

[25] Khouri MG, Douglas PS, Mackey JR, Martin M, Scott JM, Scherrer-Crosbie M, et al Cancer therapy-induced cardiac toxicity in early breast cancer: addressing the unresolved issues. Circulation. 2012;126:2749–63. doi: 10.1161/CIRCULATIONAHA.112.100560 23212997 10.1161/CIRCULATIONAHA.112.100560 PMC3667651

[26] Pinder MC, Duan Z, Goodwin JS, Hortobagyi GN, Giordano SH. Congestive heart failure in older women treated with adjuvant anthracycline chemotherapy for breast cancer. J Clin Oncol. 2007;25(25):3808–15. doi: 10.1200/JCO.2006.10.4976 17664460 10.1200/JCO.2006.10.4976

[27] Giordano S, Hortobagyi GN, Kau S, Theriault R, Bondy M. Breast cancer treatment guidelines in older women. J Clin Oncol. 2005;23:783–91. doi: 10.1200/JCO.2005.04.175 15681522 10.1200/JCO.2005.04.175

[28] Yancik R, Wesley MN, Ries LA, Havlik RJ, Edwards BK, Yates JW. Effect of age and comorbidity in postmenopausal breast cancer patients aged 55 years and older. JAMA. 2001;285:885–92. 11180731 10.1001/jama.285.7.885

[29] Chlebowski RT, Haque R, Hedlin H, Col N, Paskett E, Manson JE, et al Benefit/risk for adjuvant breast cancer therapy with tamoxifen or aromatase inhibitor use by age, and race/ethnicity. Breast Cancer Res Treat. 2015;154(3):609–16. doi: 10.1007/s10549-015-3647-1 26602222 10.1007/s10549-015-3647-1

[30] Chapman JW, Meng D, Shepherd L, Parulekar W, Ingle JN, Muss HB, et al Competing causes of death from a randomized trial of extended adjuvant endocrine therapy for breast cancer. J Natl Cancer Instit. 2008;100:252–60. 10.1093/jnci/djn014 PMC2745611 18270335

[31] Weaver KE, Aziz NM, Arora NK, Forsythe LP, Hamilton AS, Oakley-Girvan I, et al Follow-up care experiences and perceived quality of care among long-term survivors of breast, prostate, colorectal, and gynecologic cancers. J Oncol Pract. 2014;10(4):e231–e9. doi: 10.1200/JOP.2013.001175 24695901 10.1200/JOP.2013.001175 PMC4094647

[32] Weaver KE, Foraker RE, Alfano CM, Rowland JH, Arora NK, Bellizzi KM, et al Cardiovascular risk factors among long-term survivors of breast, prostate, colorectal, and gynecologic cancers: a gap in surivvorship care? J Cancer Surviv. 2013;7:253–61. doi: 10.1007/s11764-013-0267-9 23417882 10.1007/s11764-013-0267-9 PMC3756807

[33] The Women's Health Initiative Study Group. Design of the Women's Health Initiative clinical trial and observational study. Control Clin Trials. 1998;19:61–109. 9492970 10.1016/s0197-2456(97)00078-0

[34] Hays J, Hunt JR, Hubbell FA, Anderson GL, Limacher M, Allen C, et al The Women's Health Initiative recruitment methods and results. Ann Epidemiol. 2003;13:S18–S77. 14575939 10.1016/s1047-2797(03)00042-5

[35] Langer RD, White E, Lewis CR, Kotchen JM, Hendrix SL, Trevisan M. The Women's Health Initiative observational study: baseline characteristics of participants and reliability of baseline measures. Ann Epidemiol. 2003;13:S107–S21. 14575943 10.1016/s1047-2797(03)00047-4

[36] Curb JD, McTiernan A, Heckbert SR, Kooperberg C, Stanford J, Nevitt M, et al Outcome ascertainment and adjudication methods in the Women's Health Initiative. Ann Epidemiol. 2003;13:S122–S8. 14575944 10.1016/s1047-2797(03)00048-6

[37] Hankey BF, Ries LA, Edwards BK. The Surveillance, Epidemiology, and End Results Program: a national resource. Cancer Epidemiol Biomarkers Prev. 1999;8:1117–21. 10613347

[38] Chlebowski RT, Anderson G. Menopausal hormone therapy and breast cancer mortality: clinical implications. Ther Adv Drug Saf. 2015;6(2):45–56. doi: 10.1177/2042098614568300 25922653 10.1177/2042098614568300 PMC4406918

[39] Chlebowski RT, Aragaki AK, Anderson G. Menopausal hormone therapy influence on breast cancer outcomes in the Women's Health Initiative. J National Compr Canc Netw. 2015;13(7):917–24. 10.6004/jnccn.2015.0106 26150583

[40] Manson JE, Greenland P, LaCroix AZ, Stefanick ML, Mouton CP, Oberman A, et al Walking compared with vigorous exercise for the prevention of cardiovascular events in women. New Engl J Med. 2002;347:716–25. doi: 10.1056/NEJMoa021067 12213942 10.1056/NEJMoa021067

[41] Abdel-Qadir H, Austin PC, Lee DS, Amir E, Tu JV, Thavendiranathan P, et al A population-based study of cardiovascular mortality following early-stage breast cancer. JAMA Cardiol. 2017;2(1):88–93. doi: 10.1001/jamacardio.2016.3841 27732702 10.1001/jamacardio.2016.3841

[42] Haque R, Prout M, Geiger AM, Kamineni A, Thwin SS, Avila C, et al Comorbidities and cardiovascular disease risk in older breast cancer survivors. Am J Manag Care. 2014;20(1):86–92. 24512167 PMC4072034

[43] Simon MS, Wassertheil-Smoller S, Thompson CA, Ray RM, Hubbell FA, Lessin L, et al Mammogrphy interval and breast cancer mortality in women over the age of 75. Breast Cancer Res Treat. 2014;148:187–95. doi: 10.1007/s10549-014-3114-4 25261290 10.1007/s10549-014-3114-4 PMC4278588

[44] Oeffinger KC, Fontham ETH, Etzioni R, Herzig A, Michaelson JS, Shih YT, et al Breast cancer screening for women at average risk: 2015 guidline update from the American Cancer Society. JAMA. 2015;314(15):1599–614. doi: 10.1001/jama.2015.12783 26501536 10.1001/jama.2015.12783 PMC4831582

[45] Early Breast Cancer Trialists' Collaborative Group. Aromatase inhibitors versus tamoxifen in early breast cancre: patient-level meta-analysis of the randomised trials. Lancet. 2015;389:1341–52. 10.1016/S0140-6736(15)61074-1 26211827

[46] Mariotto AB, Etzioni R, Hurlbert M, Penberthy L, Mayer M. Estimation of the number of women living with metastatic breast cancer in the United States. Cancer Epidemiol, Biomarkers Prev. 2017 doi: 10.1158/1055-9965.EPI-16-0889 28522448 10.1158/1055-9965.EPI-16-0889 PMC5833304

[47] Jackson JM, Defor TA, Crain AL, Kerby T, Strayer L, Lewis CE, et al Self-reported diabetes is a valid outcome in pragmatic clinical trials and observational studies. J Clin Epidemiol. 2013;66:349–50. doi: 10.1016/j.jclinepi.2012.01.013 22564498 10.1016/j.jclinepi.2012.01.013

[48] Margolis KL, Lihong Q, Brzyski R, Bonds DE, Howard BV, Kempainen S, et al Validity of diabetes self-reports in the Women's Health Initiative: comparison with medication inventories and fasting glucose measurements. Clin Trials. 2008;5:240–7. doi: 10.1177/1740774508091749 18559413 10.1177/1740774508091749 PMC2757268

[49] Knobf MT, Coviello J. Lifestyle interventions for cardiovascular risk reduction in women with breast cancer. Curr Cardiol Rev. 2011;7:250–7. doi: 10.2174/157340311799960627 22758626 10.2174/157340311799960627 PMC3322443

[50] Montazeri K, Unitt C, Foody JM, Harris JR, Partridge AH, Moslehi J. ABCDE steps to prevent heart disease in breast cancer survivors. Circulation. 2014;130:e157–e9. doi: 10.1161/CIRCULATIONAHA.114.008820 25462826 10.1161/CIRCULATIONAHA.114.008820

[51] Singla A, Kumar G, Bardia A. Personalizing cardiovascular disease prevention among breast cancer survivors. Curr Opin Cardiol. 2012;27:515–24. doi: 10.1097/HCO.0b013e3283570040 22874128 10.1097/HCO.0b013e3283570040

