# Supplementary material for: Cardiovascular disease and mortality after breast cancer in postmenopausal women: Results from the Women’s Health Initiative
Source: PLoS One. 2017 Sep 21;12(9):e0184174. doi: 10.1371/journal.pone.0184174 (PMC5608205; doi:10.1371/journal.pone.0184174)
Supplement: S1 Table — a n = 212 for age 70–74.9, and n = 406 for age 75–79.9 at breast cancer diagnosis. (PDF) [file pone.0184174.s001.pdf]

S1 Table. Age at baseline and age at breast cancer among women with breast cancer.

		Age at Breast Cancer				
		**50-59**	**60-69**	**70-79**	**80+**	Total
**Age at Study Entry**	**50-59**	640	771			1,411
	**60-69**		1,207	884		2,091
	**70-79**			618 [a]	220	838
	Total	640	1,978	1,502	220	4,340
With Complete Staging Information		627	1,957	1,482		4,066

[a] n = 212 for age 70-74.9, and n = 406 for age 75-79.9 at breast cancer diagnosis.
