# Supplementary material for: Cardiovascular disease and mortality after breast cancer in postmenopausal women: Results from the Women’s Health Initiative
Source: PLoS One. 2017 Sep 21;12(9):e0184174. doi: 10.1371/journal.pone.0184174 (PMC5608205; doi:10.1371/journal.pone.0184174)
Supplement: S2 Table — MET indicates metabolic equivalent score. (PDF) [file pone.0184174.s002.pdf]

S2 Table. Study entry characteristics and cardiovascular disease (CVD) risk factors by CVD status in women without incident breast cancer.

	**Women without Incident Breast Cancer (n = 97,576)**		
	**No CVD since study entry n (%)**	**CVD since study entry n (%)**	**p value**
**Age at Entry**			
50 – 59 years	33,490 (39.35)	2,305 (18.48)	
60 – 69 years	38,323 (45.03)	6,215 (49.83)	
70 – 79 years	13,291 (15.62)	3,952 (31.69)	< .0001
**Race/Ethnicity**			
White	73,389 (86.42)	10,815 (86.94)	
Black	5,779 (6.81)	999 (8.03)	
Hispanic	2,669 (3.14)	297 (2.39)	
Asian/Pacific Islander	1,939 (2.28)	166 (1.33)	
American Indian	280 (0.33)	39 (0.31)	
Other	863 (1.02)	124 (1.00)	< .0001
**Education**			
Less than high school	27,778 (3.29)	681 (5.51)	
High school diploma	13,180 (15.59)	2,504 (20.27)	
Some college	30,881 (36.53)	4,852 (39.27)	

College degree or higher	37,698 (44.59)	4,319 (34.95)	< .0001
**Study Arm**			
Clinical Trial	38,268 (44.97)	6,193 (49.66)	
Observational	46,836 (55.03)	6,279 (50.34)	< .0001
**Body Mass Index (kg/m^2)**			
< 25	32,424 (38.43)	3,659 (29.6)	
25.0 – 29.9	29,484 (34.95)	4,511 (36.49)	
30.0 – 34.9	14,280 (16.93)	2,585 (20.91)	
35 – 39.9	5,444 (6.45)	1,066 (8.62)	
≥ 40	2,740 (3.25)	540 (4.37)	< .0001
**Waist Circumference**			
≤ 88 cm	55,146 (65.03)	6,587 (53.01)	
> 88 cm	29,652 (34.97)	5,840 (46.99)	< .0001
**Smoking**			
Never	44,038 (52.3)	6,011(48.77)	
Past	35,423 (42.07)	5,211 (42.28)	
Current	4,738 (5.63)	1,102 (8.94)	< .0001
**Hypertension**			
No	58,814 (72.86)	6,416 (55.01)	

Yes & untreated	5,837 (7.23)	1,180 (10.12)	
Yes & treated	16,073 (19.91)	4,067 (34.87)	< .0001
**Diabetes**			
No	82,297 (96.76)	11,340 (91)	
Yes	2,760 (3.24)	1,121 (9)	< .0001
**Hypercholesterolemia**			
No	71,522 (89.15)	9,711 (83.55)	
Yes	8,707 (10.85)	1,912 (16.45)	< .0001
**Physical Activity (total MET-hours/week)**			
< 2.5	18,761 (23.1)	3,269 (27.8)	
2.5 – 18.24	40,641 (50.04)	5,951 (50.62)	
≥ 18.25	21,815 (26.86)	2,537 (21.58)	< .0001
**Menopausal Hormone Therapy**			
Never	35,052 (41.21)	5,922 (47.54)	
Past	12,597 (14.81)	2,276 (18.27)	
Current	37,400 (43.97)	4,259 (34.19)	< .0001

MET indicates metabolic equivalent score.

S3 Table. Breast cancer characteristics by cardiovascular disease (CVD) status in women with invasive breast cancer.

	Total	No CVD		CVD after Incident Breast Cancer		p value	
**Age at Breast Cancer** Years, Mean ± SD	68.03 ± 7.19	67.8 ± 7.2		70.3 ± 6.9		< .0001	
	**n (%)**	**n**	**%**	**n**	**%**	**p** [a]	**p** [b]
**Stage**							
Localized	3,262 (75.16)	2,994	75.21	268	74.65		
Regional	987 (22.74)	902	22.66	85	23.68		
Distant	29 (0.67)	27	0.68	2	0.56		
Unknown/missing	62 (1.43)	58	1.46	4	1.11	0.92	0.89
**Tumor Size**							
≤ 5 mm	531 (12.24)	493	12.38	38	10.58		
5.1 – 10 mm	1,136 (26.18)	1,051	26.40	85	23.68		
10.1 – 20 mm	1,678 (38.66)	1,524	38.28	154	42.90		
> 20 mm	793 (18.27)	725	18.21	68	18.94		
Missing	202 (4.65)	188	4.72	14	3.90	0.38	0.30
**Positive Lymph Nodes**							
No	2,975 (68.55)	2,741	68.85	234	65.18		
Yes	941 (21.68)	861	21.63	80	22.28		
Unknown/missing	424 (9.77)	379	9.52	45	12.53	0.15	0.53
**Grade**							
Well differentiated	1,149 (26.47)	1,064	26.73	85	23.68		
Moderately differentiated	1,673 (38.55)	1,537	38.61	136	37.88		
Poorly differentiated	947 (21.82)	859	21.58	88	24.51	0.59	0.47
