# Supplementary material for: Cardiovascular disease and mortality after breast cancer in postmenopausal women: Results from the Women’s Health Initiative
Source: PLoS One. 2017 Sep 21;12(9):e0184174. doi: 10.1371/journal.pone.0184174 (PMC5608205; doi:10.1371/journal.pone.0184174)
Supplement: S3 Table — HER2 indicates human epidermal growth factor receptor 2. aChi-square test included missing category. bChi-square test did not include missing category. (PDF) [file pone.0184174.s003.pdf]

S3 Table. Breast cancer characteristics by cardiovascular disease (CVD) status in women with invasive breast cancer.

	Total	No CVD		CVD after Incident Breast Cancer		p value	
**Age at Breast Cancer** Years, Mean ± SD	68.03 ± 7.19	67.8 ± 7.2		70.3 ± 6.9		< .0001	
	**n (%)**	**n**	**%**	**n**	**%**	**p** [a]	**p** [b]
**Stage**							
Localized	3,262 (75.16)	2,994	75.21	268	74.65		
Regional	987 (22.74)	902	22.66	85	23.68		
Distant	29 (0.67)	27	0.68	2	0.56		
Unknown/missing	62 (1.43)	58	1.46	4	1.11	0.92	0.89
**Tumor Size**							
≤ 5 mm	531 (12.24)	493	12.38	38	10.58		
5.1 – 10 mm	1,136 (26.18)	1,051	26.40	85	23.68		
10.1 – 20 mm	1,678 (38.66)	1,524	38.28	154	42.90		
> 20 mm	793 (18.27)	725	18.21	68	18.94		
Missing	202 (4.65)	188	4.72	14	3.90	0.38	0.30
**Positive Lymph Nodes**							
No	2,975 (68.55)	2,741	68.85	234	65.18		
Yes	941 (21.68)	861	21.63	80	22.28		
Unknown/missing	424 (9.77)	379	9.52	45	12.53	0.15	0.53
**Grade**							
Well differentiated	1,149 (26.47)	1,064	26.73	85	23.68		
Moderately differentiated	1,673 (38.55)	1,537	38.61	136	37.88		
Poorly differentiated	947 (21.82)	859	21.58	88	24.51	0.59	0.47

Anaplastic	101 (2.33)	93	2.34	8	2.23		
Unknown/not done/missing	470 (10.83)	428	10.75	42	11.70		
**Laterality**							
Right	2,129 (49.06)	1,959	49.21	170	47.35		
Left	2,183 (50.30)	1,994	50.09	189	52.65		
Paired site/mid line tumor	5 (0.12)	5	0.13	0	0.00		
Missing	23 (0.53)	23	0.58	0	0.00	0.47	0.58
**Estrogen Receptor**							
Positive	3,405 (78.46)	3,120	78.37	285	79.39		
Negative	571 (13.16)	527	13.24	44	12.26		
Borderline	10 (0.23)	10	0.25	0	0.00		
Unknown/not done/missing	354 (8.16)	324	8.14	30	8.36	0.75	0.55
**Progesterone Receptor**							
Positive	2,820 (64.98)	2,596	65.21	224	62.40		
Negative	1,092 (25.16)	996	25.02	96	26.74		
Borderline	26 (0.60)	23	0.58	3	0.84		
Unknown/not done/missing	402 (9.26)	366	9.19	36	10.03	0.71	0.57
**HER2**							
Positive	485 (11.18)	458	11.50	27	7.52		
Negative	2,397 (55.23)	2.202	55.31	195	54.32		
Borderline	31 (0.71)	29	0.73	2	0.56		
Unknown/not done/missing	1,427 (32.88)	1,292	32.45	135	37.60	0.06	0.15

HER2 indicates human epidermal growth factor receptor 2.

[a] Chi-square test included missing category.

[b] Chi-square test did not include missing category.
