# Supplementary material for: Cardiovascular disease and mortality after breast cancer in postmenopausal women: Results from the Women’s Health Initiative
Source: PLoS One. 2017 Sep 21;12(9):e0184174. doi: 10.1371/journal.pone.0184174 (PMC5608205; doi:10.1371/journal.pone.0184174)
Supplement: S4 Table — CVD indicates cardiovascular disease. (PDF) [file pone.0184174.s004.pdf]

S4 Table. Survival status and cause of death in women with invasive breast cancer at ages 50-59 and 60-69.

	Breast Cancer Diagnosis at Age 50-59				Breast Cancer Diagnosis at Age 60-69			
**Survival status**	**Localized n (%)**	**Regional n (%)**	**Distant n (%)**	**All n (%)**	**Localized n (%)**	**Regional n (%)**	**Distant n (%)**	**All n (%)**
**Alive**	411 (92.2)	152 (86.4)	1 (20.0)	564 (90.0)	1,343 (90.8)	380 (81.7)	7 (53.9)	1,730 (88.4)
**Dead**	35 (7.9)	24 (13.6)	4 (80.0)	63 (10.0)	136 (9.2)	85 (18.3)	6 (46.1)	227 (11.6)
**Total (%)**	446 (71.1)	176 (28.1)	5 (0.8)	627 (100)	1,479 (75.6)	465 (23.8)	13 (6.6)	1,957 (100)
**Causes of Death**	**Localized n (%)**	**Regional n (%)**	**Distant n (%)**	**All n (%)**	**Localized n (%)**	**Regional n (%)**	**Distant n (%)**	**All n (%)**
**Breast Cancer**	14 (40.0)	18 (75.0)	4 (100)	36 (57.1)	34 (25.0)	56 (65.9)	4 (66.7)	94 (41.4)
**Other Major Cancers**	1 (2.9)	0 (0.0)	0 (0.0)	1 (1.6)	18 (13.2)	2 (2.4)	0 (0.0)	20 (8.8)
**Other Cancer Death**	5 (14.3)	1 (4.2)	0 (0.0)	6 (9.5)	17 (12.5)	7 (8.2)	0 (0.0)	24 (10.6)
**Total CVD**	5 (14.3)	2 (8.3)	0 (0.0)	7 (11.1)	8 (5.9)	5 (5.9)	1 (16.7)	14 (6.2)
**Coronary heart disease**	1 (2.9)	1 (4.2)	0 (0.0)	2 (3.2)	3 (2.2)	3 (3.5)	0 (0.0)	6 (2.6)
**Stroke**	2 (5.7)	0 (0.0)	0 (0.0)	2 (3.2)	2 (1.5)	0 (0.0)	0 (0.0)	2 (0.9)

**Other CVD**	2 (5.7)	1 (4.2)	0 (0.0)	3 (4.8)	3 (2.2)	2 (2.4)	1 (16.7)	6 (2.6)
**Others**	10 (28.6)	3 (12.5)	0 (0.0)	13 (20.6)	59 (43.4)	15 (17.7)	1 (16.7)	75 (33.0)

CVD indicates cardiovascular disease.
