# Supplementary material for: Cardiovascular disease and mortality after breast cancer in postmenopausal women: Results from the Women’s Health Initiative
Source: PLoS One. 2017 Sep 21;12(9):e0184174. doi: 10.1371/journal.pone.0184174 (PMC5608205; doi:10.1371/journal.pone.0184174)
Supplement: S5 Table — (PDF) [file pone.0184174.s005.pdf]

S5 Table. Details of other causes of death in women by age at localized breast cancer diagnosis and women without breast cancer by age at study entry.

	Localized Breast Cancer			No Breast Cancer		
	Age at Breast Cancer			Age at Baseline		
Causes of Death	50-59 years n (%)	60-69 years n (%)	≥ 70 years n (%)	50-59 years n (%)	60-69 years n (%)	≥ 70 years n (%)
**Total Deaths**	35 (100)	136 (100)	288 (100)	1,352 (100)	4,362 (100)	4,102 (100)
**Others**	10 (28.6)	59 (43.4)	127 (44.1)	662 (49.0)	2,163 (49.6)	2,126 (51.8)
**COPD**	0 (0.0)	3 (2.2)	9 (3.1)	46 (3.4)	206 (4.7)	178 (4.3)
**Pneumonia**	0 (0.0)	3 (2.2)	9 (3.1)	28 (2.1)	127 (2.9)	183 (4.5)
**Alzheimers' disease/Dementia**	0 (0.0)	5 (3.6)	6 (2.1)	13 (1.1)	152 (3.5)	281 (6.8)
**Accident/suicide/other injury**	1 (2.9)	3 (2.2)	7 (2.5)	45 (3.3)	136 (3.1)	137 (3.3)
**Sepsis**	1 (2.9)	3 (2.2)	3 (1.0)	41 (3.0)	103 (2.4)	105 (2.6)
**Pulmonary fibrosis**	0 (0.0)	1 (0.6)	4 (1.4)	14 (1.0)	59 (1.4)	39 (1.0)
**Renal failure**	0 (0.0)	1 (0.6)	4 (1.4)	16 (1.2)	60 (1.4)	74 (1.8)

**Hepatic cirrhosis**	0 (0.0)	1 (0.6)	3 (1.0)	13 (1.0)	39 (0.9)	14 (0.3)
**Parkinson's**	0 (0.0)	1 (0.6)	3 (1.0)	3 (0.2)	24 (0.6)	30 (0.7)
**Other causes**	8 (22.8)	39 (28.6)	79 (27.5)	403 (29.7)	1,257 (28.7)	1,085 (26.5)
