# Supplementary material for: Cardiovascular disease and mortality after breast cancer in postmenopausal women: Results from the Women’s Health Initiative
Source: PLoS One. 2017 Sep 21;12(9):e0184174. doi: 10.1371/journal.pone.0184174 (PMC5608205; doi:10.1371/journal.pone.0184174)
Supplement: S6 Table — (PDF) [file pone.0184174.s006.pdf]

S6 Table. The number of cardiovascular disease (CVD) events and death outcomes by age at breast cancer diagnosis in women with invasive breast cancer (N = 4,340).

**Age at Breast Cancer (years)**	**50-59 (n = 640)**	**60-69 (n = 1,978)**	**≥ 70 (n = 1,722)**
	**# Event (% Event)**	**# Event (% Event)**	**# Event (% Event)**
**CVD**	30 (4.69)	141 ((7.13)	188 (10.92)
**Coronary Heart Disease (CHD)**	7 (1.09)	39 (1.97)	96 (5.57)
**Myocardial Infarction**	5 (0.78)	34 (1.72)	61 (3.54)
**Angina**	6 (0.94)	21 (1.06)	10 (0.58)
**Coronary Revascularization**	14 (2.19)	65 (3.29)	58 (3.37)
**Heart Failure**	5 (0.78)	15 (0.76)	13 (0.75)
**Peripheral Arterial Disease**	3 (0.47)	8 (0.40)	8 (0.46)
**Stroke**	5 (0.78)	36 (1.82)	60 (3.48)
**Total Death**	69 (10.78)	234 (11.83)	432 (25.09)
**CVD Death**	7 (1.09)	14 (0.71)	85 (4.94)
**CHD Death**	2 (0.31)	6 (0.30)	40 (2.32)
