# Supplementary material for: Cardiovascular disease and mortality after breast cancer in postmenopausal women: Results from the Women’s Health Initiative
Source: PLoS One. 2017 Sep 21;12(9):e0184174. doi: 10.1371/journal.pone.0184174 (PMC5608205; doi:10.1371/journal.pone.0184174)
Supplement: S7 Table — CHD indicates coronary heart disease; MI, myocardial infarction; REVASC, revascularization; and PAD, peripheral arterial disease. (PDF) [file pone.0184174.s007.pdf]

S7 Table. Events rates of cardiovascular disease (CVD) and mortality by baseline (BL) age groups.

	Invasive Breast Cancer Rate per 1,000 person-years (95% CI)			Localized Breast Cancer Rate per 1,000 person-years (95% CI)			No Breast Cancer Rate per 1,000 person-years (95% CI)		
**BL Age**	**50-59**	**60-69**	**70-79**	**50-59**	**60-69**	**70-79**	**50-59**	**60-69**	**70-79**
**CVD**	3.4 (2.6-4.4)	7.8 (6.8-9.1)	13.5 (11.2-16.2)	3.5 (2.6-4.8)	7.0 (5.8-8.3)	14.0 (11.4-17.1)	3.9 (3.8-4.1)	9.3 (9.1-9.6)	17.5 (16.9-18.0)
**CHD**	1.0 (0.6-1.6)	2.8 (2.2-3.5)	7.0 (5.4-9.0)	0.8 (0.4-1.5)	2.2 (1.7-3.0)	6.9 (5.2-9.1)	1.1 (1.1-1.2)	2.7 (2.5-2.8)	5.5 (5.2-5.8)
**MI**	0.8 (0.5-1.4)	2.0 (1.5-2.7)	4.5 (3.3-6.1)	0.7 (0.4-1.4)	1.5 (1.1-2.2)	4.6 (3.2-6.5)	1.0 (0.9-1.1)	2.2 (2.1-2.3)	4.0 (3.8-4.3)
**Angina**	0.4 (0.2-0.9)	0.9 (0.6-1.4)	1.0 (0.5-2.0)	0.6 (0.3-1.2)	0.8 (0.5-1.3)	0.8 (0.4-1.9)	0.7 (0.7-0.8)	1.6 (1.5-1.7)	2.4 (2.2-2.6)
**Coronary REVASC**	1.6 (1.1-2.3)	3.3 (2.6-4.1)	4.0 (2.9-5.6)	1.6 (1.0-2.5)	2.9 (2.2-3.8)	4.2 (2.9-6.0)	1.6 (1.5-1.7)	3.7 (3.5-3.8)	5.0 (4.7-5.3)
**PAD**	0.4 (0.2-0.8)	0.4 (0.2-0.7)	0.5 (0.2-1.2)	0.4 (0.2-1.0)	0.3 (0.1-0.7)	0.6 (0.2-1.5)	0.2 (0.2-0.2)	0.5 (0.5-0.6)	0.8 (0.7-0.9)
**Stroke**	0.9 (0.5-1.5)	2.0 (1.5-2.7)	4.5 (3.3-6.1)	0.8 (0.4-1.5)	2.0 (1.5-2.8)	4.8 (3.5-6.8)	0.8 (0.7-0.9)	2.2 (2.1-2.3)	4.6 (4.3-4.9)
**Total Death**	8.2 (7.0-9.7)	14.2 (12.8-15.8)	28.9 (25.6-32.6)	5.8 (4.6-7.3)	11.3 (9.8-12.9)	25.2 (21.7-29.1)	3.4 (3.2-3.5)	8.4 (8.3-8.6)	20.4 (19.9-20.8)
**CVD**	0.5 (0.3-1.0)	1.4 (1.0-2.0)	7.1 (5.5-9.1)	0.5 (0.2-1.1)	1.4 (0.9-2.0)	7.1 (5.4-9.4)	0.7 (0.6-0.7)	2.0 (1.9-2.1)	6.9 (6.6-7.2)

**Death**									
**CHD Death**	0.1 (0.0-0.5)	0.8 (0.5-1.2)	3.2 (2.2-4.6)	0.1 (0.0-0.6)	0.7 (0.4-1.2)	2.8 (1.8-4.3)	0.3 (0.3-0.4)	1.0 (0.9-1.0)	3.0 (2.8-3.2)

CHD indicates coronary heart disease; MI, myocardial infarction; REVASC, revascularization; and PAD, peripheral arterial disease.
