# Supplementary material for: Cardiovascular disease and mortality after breast cancer in postmenopausal women: Results from the Women’s Health Initiative
Source: PLoS One. 2017 Sep 21;12(9):e0184174. doi: 10.1371/journal.pone.0184174 (PMC5608205; doi:10.1371/journal.pone.0184174)
Supplement: S8 Table — HR indicates hazard ratio; CI, confidential interval; and MET, metabolic equivalent score. (PDF) [file pone.0184174.s008.pdf]

S8 Table. Associations between baseline risk factors and coronary heart disease (CHD) in women without breast cancer.

No Breast Cancer (n = 97,576)				
**Baseline Risk Factors**	**Total N**	**CHD Incidence n (%)**	**Age-adjusted Rate per 1,000 Person-years (95% CI)**	**Age-adjusted HR (95% CI)**
**Current Smoking**				
No	90,683	3,600 (3.97)	2.56 (2.42, 2.70)	1.00
Yes	5,840	416 (7.12)	5.31 (4.46, 6.34)	2.38 (2.15, 2.64)
**Hypertension**				
No	65,230	2,203 (3.21)	2.16 (2.01, 2.32)	1.00
Yes	27,157	1,830 (6.46)	3.93 (3.64, 4.26)	1.82 (1.71, 1.93)
**Diabetes**				
No	93,637	3,653 (3.90)	2.54 (2.41, 2.68)	1.00
Yes	3,881	415 (10.69)	6.90 (5.84, 8.15)	2.75 (2.49, 3.05)
**Hypercholesterolemia**				
No	81,233	3,226 (3.97)	2.63 (2.48, 2.78)	1.00
Yes	10,619	597 (5.62)	3.33 (2.90, 3.84)	1.23 (1.13, 1.34)
**Body Mass Index (kg/m^2)**				
< 25	36,083	1,192 (3.30)	2.12 (1.93, 2.33)	1.00
25.0 – 29.9	33,995	1,469 (4.32)	2.73 (2.51, 2.98)	1.29 (1.20, 1.40)

30.0 – 34.9	16,865	829 (4.92)	3.26 (2.91, 3.67)	1.58 (1.44, 1.72)
35 – 39.9	6,510	344 (5.28)	3.73 (3.10, 4.48)	1.85 (1.64, 2.08)
≥ 40	3,280	193 (5.88)	4.36 (3.39, 5.66)	2.33 (2.00, 2.71)
**Waist Circumference**				
≤ 88 cm	61,733	1,056 (4.79)	2.24 (2.09, 2.41)	1.00
> 88 cm	35,492	1,929 (5.44)	3.53 (3.27, 3.81)	1.63 (1.53, 1.73)
**Physical Activity (total MET-hours/week)**				
< 2.5	22,030	1,056 (4.79)	3.26 (2.94, 3.61)	1.00
2.5 – 18.24	46,592	1,967 (4.22)	2.70 (2.51, 2.91)	0.82 (0.76, 0.88)
≥ 18.25	24,352	839 (3.45)	2.21 (1.97, 2.47)	0.66 (0.61, 0.73)

HR indicates hazard ratio; CI, confidential interval; and MET, metabolic equivalent score.
