# Supplementary material for: Cardiovascular disease and mortality after breast cancer in postmenopausal women: Results from the Women’s Health Initiative
Source: PLoS One. 2017 Sep 21;12(9):e0184174. doi: 10.1371/journal.pone.0184174 (PMC5608205; doi:10.1371/journal.pone.0184174)
Supplement: S1 Fig — BC indicates breast cancer; and FU, follow up. (PPTX) [file pone.0184174.s009.pptx]

## Slide 1
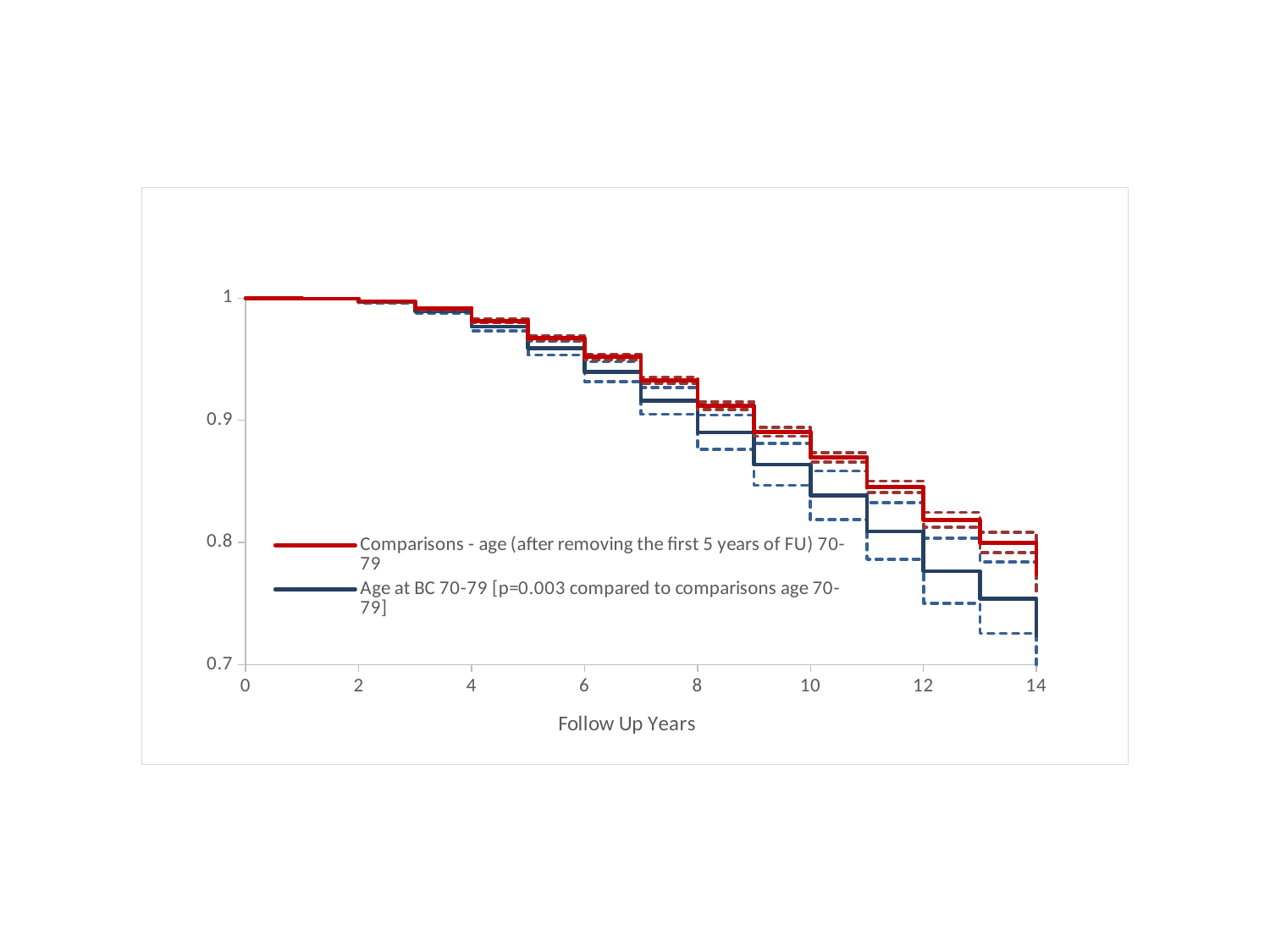

### Chart
| Category | Comparisons - age (after removing the first 5 years of FU) 70-79 | | | Age at BC 70-79 [p=0.003 compared to comparisons age 70-79] | | |
|---|---|---|---|---|---|---|
