# Supplementary material for: Cardiovascular disease and mortality after breast cancer in postmenopausal women: Results from the Women’s Health Initiative
Source: PLoS One. 2017 Sep 21;12(9):e0184174. doi: 10.1371/journal.pone.0184174 (PMC5608205; doi:10.1371/journal.pone.0184174)
Supplement: S2 Fig — BC indicates breast cancer; and FU, follow up. (PPTX) [file pone.0184174.s010.pptx]

## Slide 1
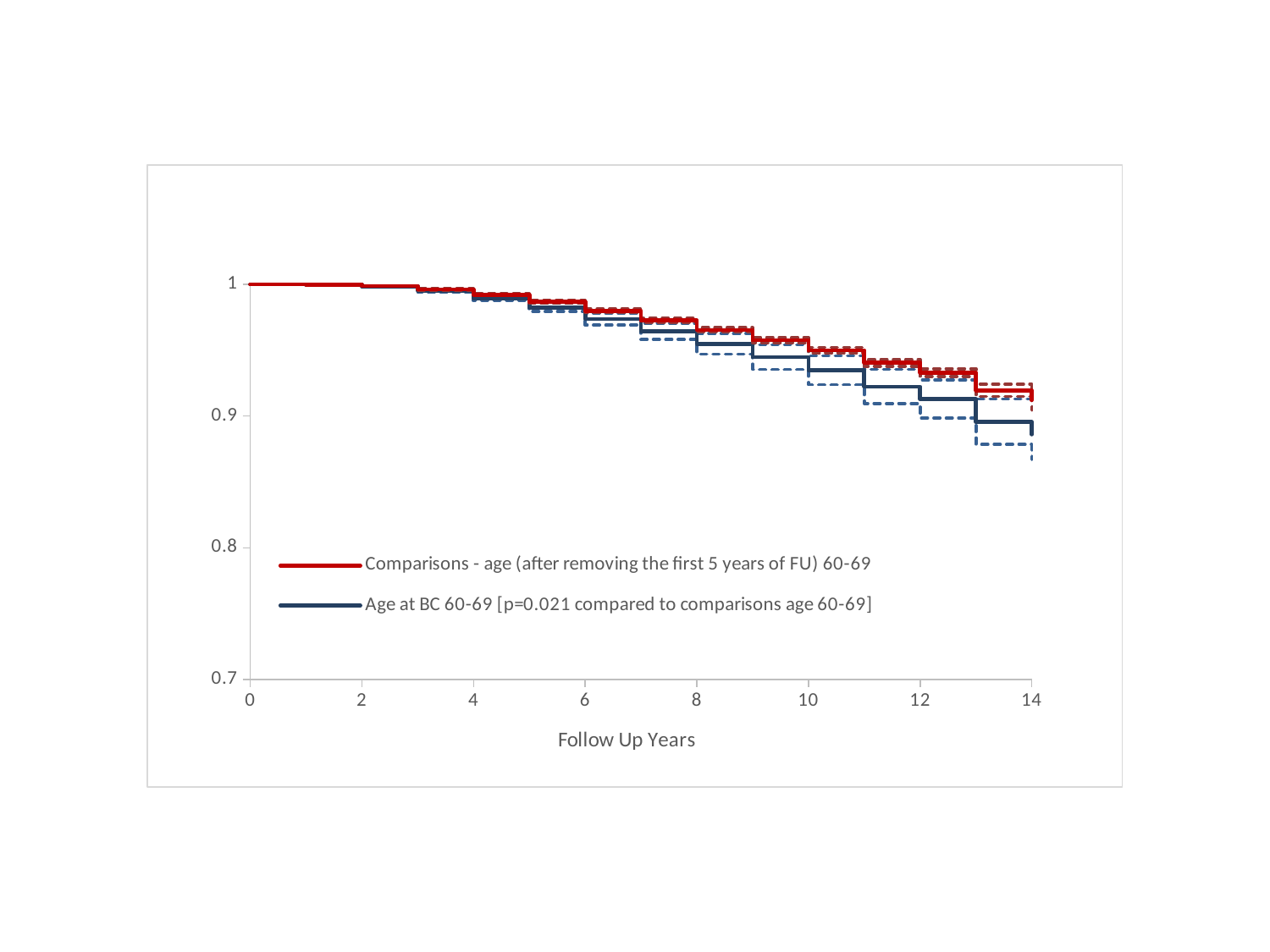

### Chart
| Category | Comparisons - age (after removing the first 5 years of FU) 60-69 | | | Age at BC 60-69 [p=0.021 compared to comparisons age 60-69] | | |
|---|---|---|---|---|---|---|
