# Supplementary material for: Cardiovascular disease and mortality after breast cancer in postmenopausal women: Results from the Women’s Health Initiative
Source: PLoS One. 2017 Sep 21;12(9):e0184174. doi: 10.1371/journal.pone.0184174 (PMC5608205; doi:10.1371/journal.pone.0184174)
Supplement: S3 Fig — BC indicates breast cancer; and FU, follow up. (PPTX) [file pone.0184174.s011.pptx]

## Slide 1
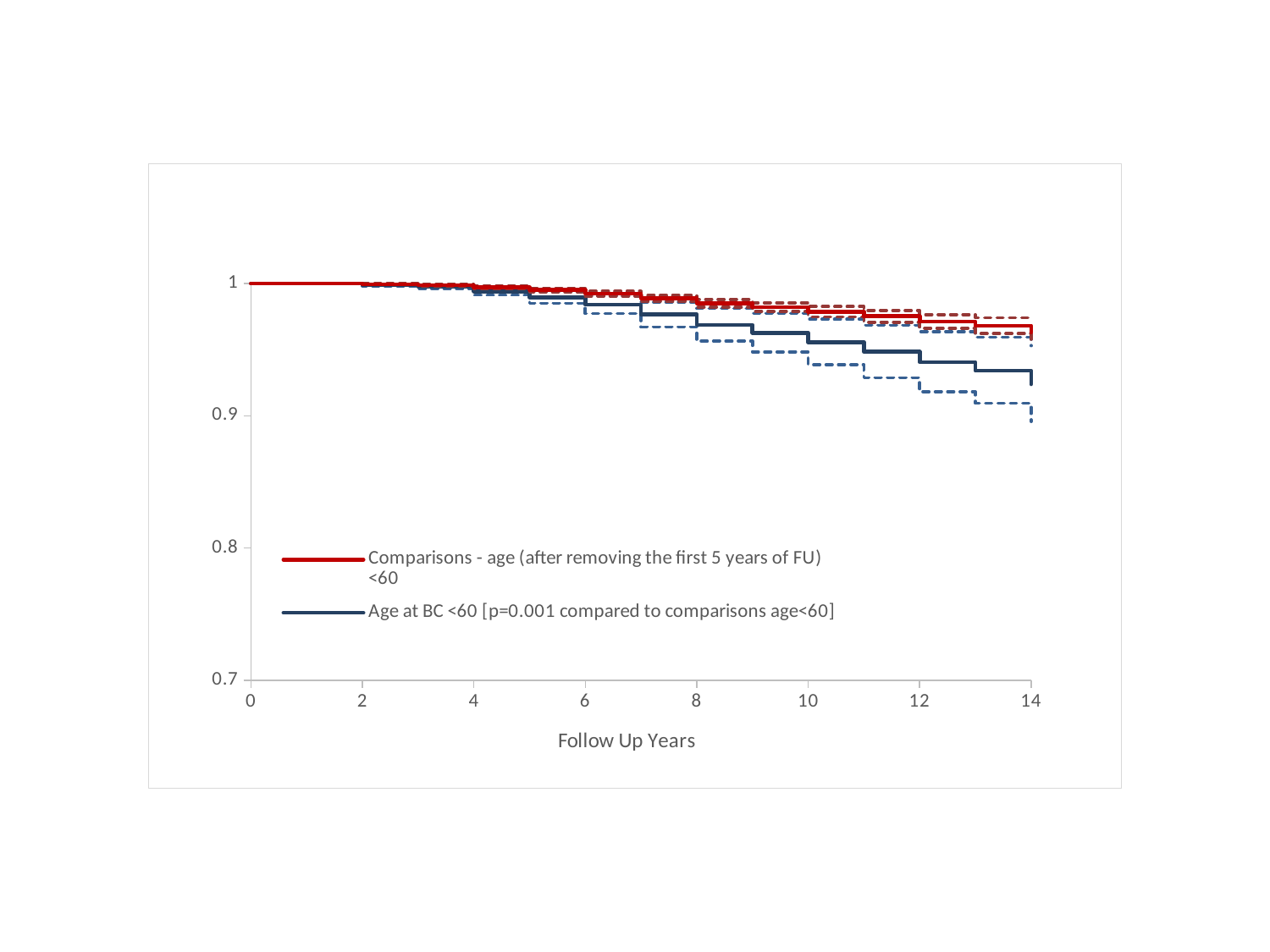

### Chart
| Category | Comparisons - age (after removing the first 5 years of FU) <60 | | | Age at BC <60 [p=0.001 compared to comparisons age<60] | | |
|---|---|---|---|---|---|---|
